# CAGE-defined promoter regions of the genes implicated in Rett Syndrome

**DOI:** 10.1186/1471-2164-15-1177

**Published:** 2014-12-24

**Authors:** Morana Vitezic, Nicolas Bertin, Robin Andersson, Leonard Lipovich, Hideya Kawaji, Timo Lassmann, Albin Sandelin, Peter Heutink, Dan Goldowitz, Thomas Ha, Peter Zhang, Annarita Patrizi, Michela Fagiolini, Alistair RR Forrest, Piero Carninci, Alka Saxena, The FANTOM Consortium

**Affiliations:** Omics Science Center, RIKEN Yokohama Institute, Omics Science Center (OSC), 1-17-22 Suehiro cho, Tsurumi ku, Yokohama, Japan; Department of Cell and Molecular Biology (CMB), Karolinska Institutet, Stockholm, Sweden; The Bioinformatics Center, Department of Biology and Biotech Research and Innovation Center, University of Copenhagen, Copenhagen, Denmark; Division of Genomic Technologies (DGT), RIKEN Center for Life Science Technologies, Yokohama, Japan; Cancer Science Institute of Singapore, National University of Singapore, Singapore, Singapore; Center for Molecular Medicine and Genetics, Wayne State University, Detroit, MI USA; Department of Neurology, School of Medicine, Wayne State University, Detroit, MI USA; RIKEN Preventive Medicine and Diagnosis Innovation Program (PMI), Wako, Japan; Telethon Kids Institute, The University of Western Australia, Perth, Australia; German Center for Neurodegenerative Diseases (DZNE), Tübingen, Germany; Eberhard Karls University, Tübingen, Germany; Centre for Molecular Medicine and Therapeutics, Child and Family Research Institute, Dept of Medical Genetics, University of British Columbia, Vancouver, Canada; FM Kirby Neurobiology Center, Department of Neurology, Boston Children’s Hospital, Harvard Medical School, Boston, MA USA; Biomedical Research Centre at Guy’s and St Thomas’ Trust, Genomics Core Facility, Guy’s Hospital, London, UK

**Keywords:** Rett Syndrome, CAGE, Transcriptomics, Promoter architecture

## Abstract

**Background:**

Mutations in three functionally diverse genes cause Rett Syndrome. Although the functions of Forkhead box G1 (*FOXG1),* Methyl CpG binding protein 2 (*MECP2)* and Cyclin-dependent kinase-like 5 (*CDKL5)* have been studied individually, not much is known about their relation to each other with respect to expression levels and regulatory regions. Here we analyzed data from hundreds of mouse and human samples included in the FANTOM5 project, to identify transcript initiation sites, expression levels, expression correlations and regulatory regions of the three genes.

**Results:**

Our investigations reveal the predominantly used transcription start sites (TSSs) for each gene including novel transcription start sites for *FOXG1*. We show that *FOXG1* expression is poorly correlated with the expression of *MECP2* and *CDKL5*. We identify promoter shapes for each TSS, the predicted location of enhancers for each gene and the common transcription factors likely to regulate the three genes. Our data imply Polycomb Repressive Complex 2 (PRC2) mediated silencing of *Foxg1* in cerebellum.

**Conclusions:**

Our analyses provide a comprehensive picture of the regulatory regions of the three genes involved in Rett Syndrome.

**Electronic supplementary material:**

The online version of this article (doi:10.1186/1471-2164-15-1177) contains supplementary material, which is available to authorized users.

## Background

Rett Syndrome (RTT) is a disorder caused by mutations in Methyl CpG binding protein 2 (*MECP2*), Forkhead box G1 (*FOXG1*) or Cyclin-dependent kinase-like 5 (*CDKL5*) genes [1–3] Although the phenotype of patients with mutations in *MECP2* differs from the phenotype of patients with *FOXG1* or *CDKL5* mutations, there are some similarities in the clinical profile that overlap with RTT. Classic RTT patients with *MECP2* mutations have a normal period of development followed by regression of acquired skills, deceleration of head circumference, epilepsy, hand stereotypies, breathing abnormalities, inability to walk or talk and intellectual disability while patients with atypical RTT may show some but not all features of classic Rett syndrome [4]. Mutations in *FOXG1* are known to cause the congenital variant of Rett syndrome where the initial normal developmental window is absent [2]. *CDKL5* mutations are found in patients with severe epilepsy during early childhood that later show features that resemble atypical RTT syndrome [5].

MeCP2 is an X-linked methyl CpG binding protein which binds methylated and unmethylated DNA [6–9] and functions as a repressor and activator of genes [10–13]. Even though *MECP2* is expressed ubiquitously [14], *MECP2* mutations and copy number variations in humans lead to neurological phenotypes such as classic or atypical Rett syndrome and in rare cases Angelman Syndrome, X-linked mental retardation and Autism (reviewed in [15]) suggesting a distinct role for MeCP2 protein in the brain [16, 17]. The level of MeCP2 protein in neurons increases with neuronal maturity [18] and it is abundantly expressed in the mature brain, almost equivalent to Histone H1 levels [19], but the level of *MECP2* mRNA in cells is reported to not correlate with the level of MeCP2 protein in cells [18].

FOXG1 protein is a brain specific member of the forkhead transcription factor family with a role in transcriptional repression. Similar to other members of the forkhead family, FOXG1 has a defined binding sequence motif [20], which bears sequence similarity to other forkhead protein binding sites.

CDKL5 protein is a serine threonine kinase, whose expression is low in embryonic stages, but increases in postnatal stages up to postnatal day 15 [21] CDKL5 mRNA is expressed in brain and all other tissues [22, 23]. CDKL5 protein levels are known to coincide with its mRNA levels [24].

Even though these three genes have different expression patterns, distinct functions and specific regulatory targets, their paths appear to intersect. Both MeCP2 and FOXG1 proteins regulate transcription via DNA binding and association with other transcriptional regulators [6–13][25, 26]. The functions of MeCP2 and CDKL5 proteins also appear to be interconnected. MeCP2 has multiple phosphorylation sites [27] and is a target of CDKL5 phosphorylation. Additionally, there are contradictory reports on the expression level of CDKL5 protein and mRNA in the absence of MeCP2 [28–30]. Altogether, these observations suggest that the overlapping features in Rett syndrome may be caused by impairment of common or intersecting biological pathways downstream of expression in the brain. Alternately, these genes may be interdependent on each other for expression or regulation, which may lead to the overlap in phenotypic features.

Although we have some knowledge of their downstream intersecting functions, we are yet unaware of the common genomic features between these three genes, which may provide insights into their regulation. Importantly, although both *MECP2* and *CDKL5* genes are expressed ubiquitously, their mutations cause a brain specific phenotype suggesting that their expression level, transcription regulation, or function in brain may be distinct from that in other tissues. We tried to resolve these questions through bioinformatics analyses using the FANTOM5 dataset [31].

Data from FANTOM5 provide the unprecedented opportunity to identify the transcription start sites (TSSs) of these genes and study their expression profile in hundreds of mouse and human samples using Cap Analysis of Gene Expression method (CAGE) [31]. In conjunction with the recently released ENCODE dataset [32]. FANTOM5 data also enable the identification of regulatory histone marks at TSSs. Since the RTT phenotype is reflected in the *Mecp2* KO mouse model [33] and studies on this disorder are conducted in mouse tissues and cells, we also included mouse samples in our analyses. We analyzed the TSS expression data from the FANTOM5 project using over 1000 human and over 450 mouse samples to identify common and diverse features of the genomic architecture of the three genes implicated in RTT (for a complete list of samples Additional file 1: Table S1). For our investigation, we divided the human and mouse samples into tissues, primary cells and cell lines to study the expression levels of the TSS of the three genes in various samples. Our data reveal the precise initiation sites for the three genes, including previously unknown TSSs for *FOXG1* in mouse and humans. We show that each of these genes use the same TSS in most tissues and provide information on the expression level of the three genes over development in multiple human and mouse samples. Although we did not find a significant correlation between the expression levels of the three genes in the brain, our genome wide analyses uncovered common transcription factors regulating the three genes, suggesting an additional molecular layer in the pathogenesis of Rett Syndrome. The FANTOM5 CAGE dataset also allowed us to locate putative enhancers regulating the three genes in human (methods described in Anderson et al., [34]) and using mouse ENCODE ChIP-seq data, we identified genomic regions bearing promoter and enhancer marks. This work is part of the FANTOM5 project. Data downloads, genomic tools and co-published manuscripts are summarized here: http://fantom.gsc.riken.jp/5/.

## Methods

### FANTOM5 samples

Single molecule CAGE profiles were generated from RNA obtained from a collection of 573 human primary cell samples (~3 donors for most cell types) covering most mammalian cell steady states. This data set was complemented with profiles of 250 different cancer cell lines, 152 human post-mortem tissues and 456 mouse samples (detailed sample list is available in Additional file 1: Table S1 and origin of each sample is available as Supplementary Material in Forrest et al. 2014 [31]). Primary cells for neurons and astrocytes discussed in this manuscript were obtained from ScienCell Research Laboratories. Human neurons were isolated from the human brain, cryopreserved at primary cultures and delivered frozen. Human astrocytes were isolated from cerebral cortex and cerebellum. Both were cryopreserved at passage one and delivered frozen.

All human samples used in the project were either exempted material (available in public collections or commercially available), or provided under informed consent. All non-exempt material is covered under RIKEN Yokohama Ethics applications (H17-34 and H21-14). Mouse tissue samples were collected as per RIKEN Yokohama institutional guidelines. Mouse primary cells were collected as per our collaborators Institutional guidelines and shipped as either purified RNA or as guanidinium isothyocyanate lysates (Trizol, Isogen or Qiazol) which were then purified using the miRNeasy kit (QIAGEN). More detailed information for each specific sample is available in Additional file: 1 Table S1 of [31].

All the data published by the Fantom5 project and by this study are available through the Fantom5 portal http://fantom.gsc.riken.jp/5/data/. All CAGE data has been deposited at DDBJ DRA under accession number DRA000991.

### Identifying CAGE derived transcription start sites

We used the FANTOM5 database ([31]) to identify transcription start sites (TSS) for our genes, using the decomposition peak identification (DPI) clustering and nomenclature developed for the FANTOM5 project [31]. We selected robust CAGE defined DPI clusters falling inside the RefSeq regions known to be associated to the three genes. To select for genuine TSSs we used the FANTOM5 TSS classifier and restricted our TSS selection to those with a value of 0.1 and above [31]. The TSSs were annotated using the names assigned to clusters in the FANTOM5 Resource browser (SSTAR, Semantic catalogue of, samples, transcription initiations, and regulations, http://fantom.gsc.riken.jp/5/sstar/). Annotation files were built in the context of the FANTOM5 project with respect to Gencode v10 gene model (human), RefSeq (mouse), CpG islands and TATA box in bed format.

### TSS expression

We extracted expression information for each TSSs using the FANTOM5 expression dataset for tissues, cell lines and primary cells in human and mouse (see Additional file 1: Table S1 for a full list of samples and TPM expressions). The expression values are shown in tags per million (TPM) calculated on a per-library total expression. We discarded all the TSSs that did not have over 5 TPM expression in any of the samples. All expression level figures, heatmaps and correlations were calculated using R (http://www.r-project.org/).

### Mecp2 and histone expression comparison

We extracted the CAGE defined promoters associated to the genes whose products form the Histone1 transcripts (*HIST1H1A*, *HIST1H1B*, *HIST1H1C*, *HIST1H1D*, *HIST1H1E*, *H1F0*, *H1FX*). All the values for different genes were added together and compared to expression levels of *MECP2*.

### Identifying TSS overlaps with ChIP seq data from human and mouse ENCODE TSSs

TSSs identified were expanded by 500 nucleotides on either side (±500bp). ChIP seq data from Human and mouse ENCODE were downloaded as bed files and intersected with our expanded TSS using intersectBed [35].

### Defining human enhancers

To identify enhancers associated with the human Rett genes, we used the CAGE derived enhancer database from Andersson et al. [34]. In short, the identified enhancers from Andersson et al. within 500kb distance from the identified Rett genes promoters were selected [34]. The expression of pairs of enhancers and promoters was then compared in all the human samples using a Pearson correlation test. The resulting comparisons were then corrected for multiple testing using Bonferroni correction. Only enhancers significantly correlated (corrected *P* < 0.05) with any of the three Rett genes promoters were included.

### Transcription factor binding site (TFBS) analysis

We downloaded the whole-genome alignment of the human genome with 45 other vertebrate genomes, and of the mouse genome with 29 other vertebrate genomes, from the UCSC Genome Browser database [36]. From these alignments, we retained the alignments between the human, macaque, mouse, rat, cow, horse, dog, opossum, and chicken genomes only, and used the T-Coffee alignment tool [37] on 1000 bp segments of the genome to optimize the alignment for the nine selected genomes. We then ran MotEvo [38] on these whole-genome alignments using a background prior probability of 0.98, a prior for the UFE (unidentified functional element) model of 200 relative to the weight matrices, a UFE motif length of 8 base pairs, and a uniform background sequence probability. A posterior probability calculated by MotEvo for a putative TFBS was retained if it was at least 0.2. We used the center position for a given CAGE promoter on the genome as a reference point, and summed the posterior probabilities for the putative binding sites for each transcription factor within a distance of 500 basepairs of the reference point to obtain the estimated number of binding sites for each transcription factor. To evaluate the statistical significance of this number, for each transcription factor we estimated the number of binding sites in exactly the same way for all 184,827 (human) or 116,227 (mouse) promoters in the FANTOM5 data sets, and ranked the promoters accordingly. The tail probability was then obtained by dividing the rank of the promoter of interest by 184,827 (human) or 116,227 (mouse).

## Results

### *FOXG1*expression in mouse and humans

**Figure 1:**
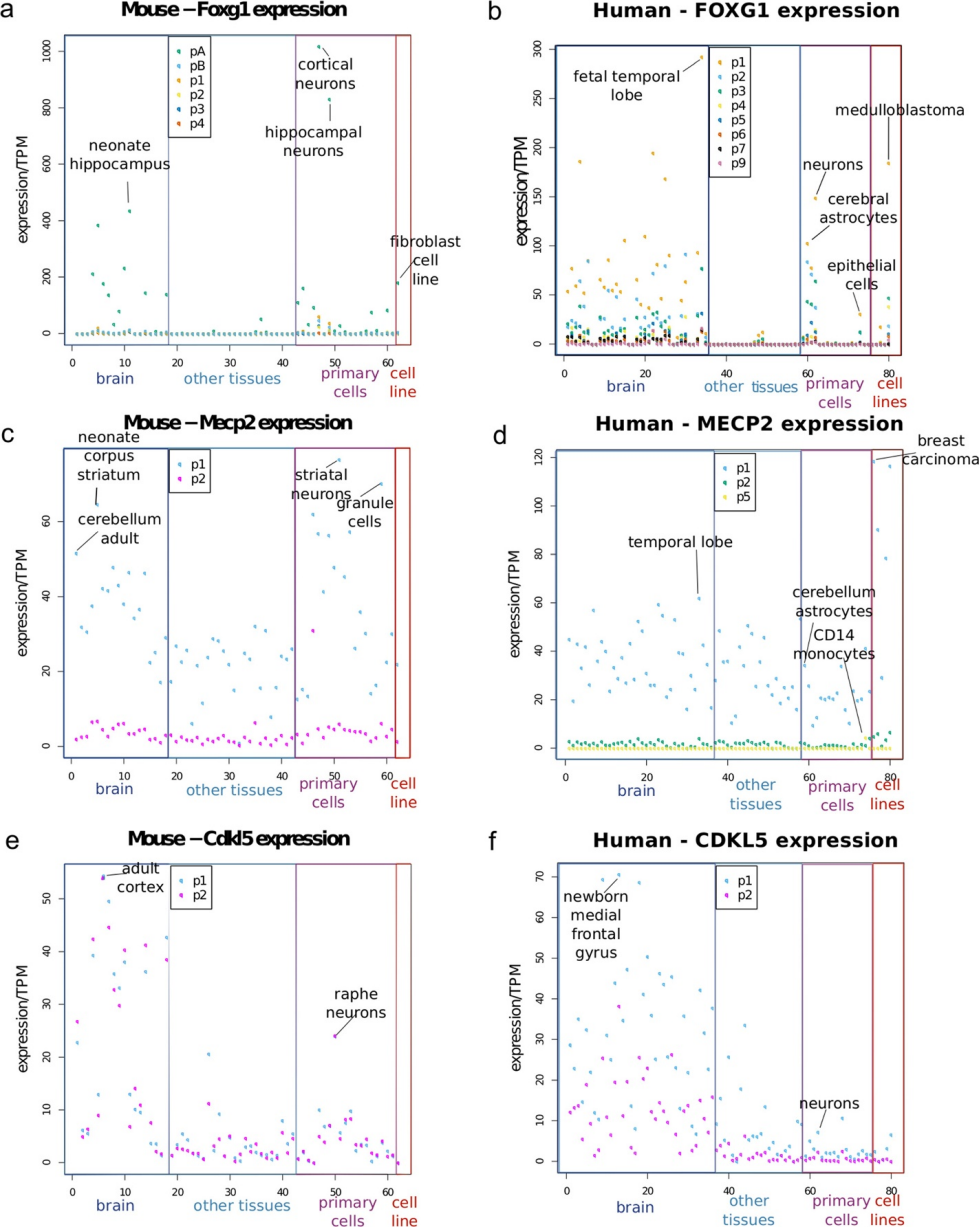
**Expression levels of the identified TSS for the three genes.** Dot plots showing the expression level of each promoter in TPM values in all brain regions, and selected other samples (based on expression level). The novel promoter pA@Foxg1 is the most highly expressed *Foxg1* TSS in mouse primary cells and brain tissue **(a)**, with the highest expression in cortical neurons (1018 TPM) and neonate hippocampus (435 TPM). Among mouse cells, we find high levels of p1@ Foxg1 expressed in hippocampal neurons and fibroblast cell line. In human samples **(panel b)** the highest expression of *FOXG1* is seen from p1@FOXG1 in fetal temporal lobe (292 TPM), among primary cells in neurons (149 TPM) and among cell lines in medulloblastoma cell line (184 TPM). For mouse *Mecp2*, the highest expression of p1@Mecp2 is in striatal neurons (77 TPM) and cerebellar granule cells (70 TPM) and among mouse tissues **(panel c)** the maximum expression is seen in neonate corpus striatum (65 TPM) and adult cerebellum (52 TPM). For human, the highest expression of p1@MECP2 is found in cancer cell lines including breast carcinoma cell line (119 TPM) **(panel d)**. In human brain the highest expression of p1@MECP2 is found in the temporal lobe (63 TPM). The two promoters of *Cdkl5* in mouse are co-expressed with highest expression in adult cortex in the brain and raphe neurons among primary cells **(panel e)**. In humans **(panel f)** the two promoters are expressed differentially with transcripts arising from p1 over-represented. p1@CDKL5 expression is highest in the newborn medial frontal gyrus and in neurons. In human cancer cell lines, *CDKL5* is generally expressed at low levels (less than 10 TPM) from either of the promoters (p1 > p2), with a few exceptions (Additional file 1: Table S1, Additional file 3: Figure S1f).

**Figure 2:**
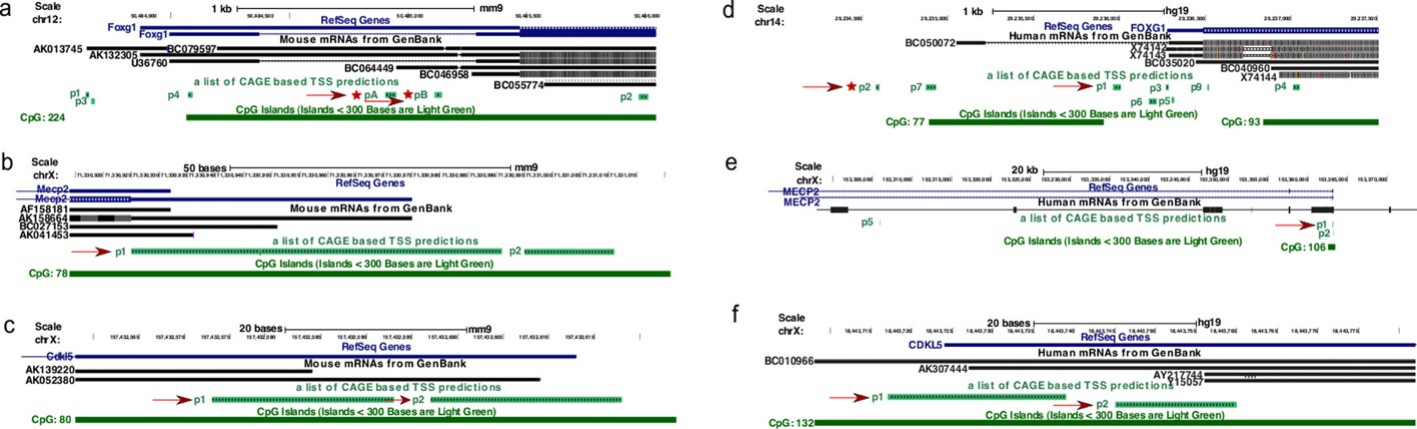
**Locations of the TSSs identified for the three genes.** Genome browser images showing all the TSSs identified in this study for *FOXG1* **(panels a and d)**, *MECP2* **(panels b and e)** and *CDKL5* **(panels c and f)** in mouse **(panels a,b and c)** and humans **(panels d, e and f)**. In each panel the top two tracks show RefSeq genes and mRNAs from Genbank. The third track shows FANTOM5 TSS and the bottom track shows CpG islands. Red arrows mark the key TSSs for each gene. We found 6 TSSs for *Foxg1* in mouse **(panel a)** and 8 TSSs in humans **(panel d)**. Novel TSSs are identified by asterisks. We also found a CD14 specific intronic TSS p5 for *MECP2* in human cells.

Analyses of TSS from 1193 human samples and 457 mouse samples comprised of tissues, primary cells and cell lines (only one mouse cell line was investigated in FANTOM5) identified 8 TSSs for human *FOXG1* and 6 TSSs for mouse *Foxg1* (Additional file 2: Table S2). *FOXG1* expression above 1 TPM was found in 23% (231) and 30% (140) of human and mouse samples respectively, suggesting that the expression of this gene was limited to selected tissues (Additional file 1: Table S1). Transcription start sites were defined as novel if they were found at a distance of over 500 bp from the known RefSeq TSSs. Our data show 3 TSSs in mouse highly expressed in brain sub-regions and cells, two of which are novel. The expression levels of different TSSs of *FOXG1* were variable in human and mouse samples, with the highest expression seen in specific regions of the brain (Figures 1a, b, Additional file 3: Figures S1a,b). The top three initiation sites were located at (in order of their expression levels) chr12:50484904..50484950,+; (pA@Foxg1, novel promoter, located more than 1000 bases downstream of the RefSeq annotated TSSs) chr12:50483639..50483654,+; (p1@Foxg1, 200 bp upstream of the two annotated *Foxg1* TSSs) and chr12:50485112..50485144,+;(pB@Foxg1, novel promoter, 1200 bp downstream from the annotated RefSeq initiation sites) (Figure 2a). Expression of mouse *Foxg1* was also restricted to brain tissue and brain related cells, but surprisingly the two novel TSSs of mouse *Foxg1* were also found highly expressed in the single mouse cell line sequenced in the FANTOM5 project (fibroblast cell line) suggesting that other than the brain, fibroblast cell lines may be useful for *in vitro* analysis of *Foxg1* in mouse (Figure 1a).

In human, there is RefSeq annotation support for a single *FOXG1* isoform and therefore a single TSS. We found 8 TSSs for human *FOXG1* expressed over 5 TPM and the 3 TSSs with the highest expression in human brain were located at chr14:29235961..29236008,+ (p1@FOXG1); chr14:29234581..29234601,+ (p2@FOXG1; novel); and chr14:29236269..29236285,+ (p3@FOXG1), with distances of 317, 1697 and 9 bases upstream of the RefSeq annotated TSS, respectively (Additional file 2: Table S2, Figure 2d, Additional file 3: Figure S1). Thus, our analyses reveal that in human brain the highest used TSS for *FOXG1* is located 317 bases upstream of the annotated start site. Contrary to mouse, where we found expression level differences of over 10-fold between TSSs, in human samples, the difference in expression between the three TSSs was less than 2-fold (Figure 1a, 1b, Additional file 1: Table S1).

Intriguingly, we did not find *FOXG1* expression in mouse or human cerebellum suggesting silencing of *FOXG1* in cerebellum. Inability to detect TSS expression may result from technical artifacts such as low expression levels not discernible at the conducted depth of sequencing or the use of an alternate tissue specific start site. To rule out technical artifacts, we referred to the ENCODE dataset to investigate signs of transcriptional activity in the chromosomal location of *Foxg1* and up to 10 kb upstream in mouse cerebellum. We analyzed ENCODE data for DNAse-I hypersensitive sites (DNAse-I HSS), which are known to faithfully recognize active transcription initiation sites [39], in mouse cerebellum, cerebrum and whole brain. Our analyses revealed an absence of DNAse-I HSS in mouse cerebellum, while DNAse-I HSS were present in cerebrum and whole brain samples (Additional file 4: Figure S2). Since DNAse-I HSS usually coincide with the active promoter specific histone mark of trimethylated Histone 3 lysine 4 (H3K4me3) [39] and transcriptionally active enhancers may also bear the specific histone mark of acetylated Histone 3 lysine 27 (H3K27ac), we looked for these two marks in mouse cerebellum and mouse cortex. We found that H3K4me3 and H3K27ac were enriched at the locus in 8-week old cortex samples, but not in 8-week old cerebellum samples. Surprisingly, our investigations revealed trimethylated Histone 3 lysine 27 (H3K27me3) enrichment at this chromosomal locus in mouse cerebellum, suggesting silencing of *Foxg1* by Polycomb Repressive Complex 2 (PRC2) (Additional file 4: Figure S2). Since ChIP data for active and repressive histone marks in human brain is not available from ENCODE at this time, we were unable to confirm similar chromatin signatures for PRC2 silencing of *FOXG1* in the human cerebellum.

Silencing by chromatin remodeling proteins such as PRC2 requires a non-coding RNA to mediate chromatin modification [40]. Therefore, we searched for potential cis-regulatory ncRNAs that may mediate *Foxg1* silencing. We found one ncRNA downstream of *Foxg1* (RefSeq NR_026733), however its expression was not entirely discordant with that of *Foxg1* (data not shown). Analysis of the ncRNA database and manual annotation of UCSC Genome Browser revealed several ncRNAs within a genomic window of 1.5 MB around *Foxg1*, but none of the listed ncRNAs were detectable in the FANTOM5 CAGE dataset.

### *MECP2*expression in mouse and humans

In humans and mouse we identified two TSSs for *MECP2*, less than 100 bases upstream of the RefSeq annotated start sites of which p1@MECP2/Mecp2 was expressed predominantly in most tissues and p2@MECP2/Mecp2 displayed a stable low level expression in all tissues (expression less than 10 TPM) (Additional file 2: Table S2, Figures 1c, 1d, 2b and 2e). We found an additional intronic promoter (p5@MECP2) in humans alone, expressed exclusively in blood primary cells, particularly in CD14 monocytes (Figure 1d and Figure 2e). Expression of p1@MECP2 in humans and mouse was found above 5 TPM in most tissues, primary cells and cell lines, suggesting that transcripts arising from this promoter were ubiquitously expressed. Surprisingly our analysis of human tissues and cells revealed that the highest expression of *MECP2* was seen in non-neuronal tissues (vagina and ovary) and cell lines (Breast Carcinoma, Krukenberg tumour, lens epithelial and lung adenocarcinoma) (Additional file 3: Figure S1d). In agreement with previous reports in mouse, we found that at mRNA level, the expression of p1@Mecp2 in astrocytes (15 TPM) was much lower than in neurons (77 to 41 TPM) (Additional file 3: Figure S1c) but among human primary cells, the expression of p1@MECP2 in neurons (12 TPM) was lower than the p1@MECP2 expression in astrocytes (34 TPM). In contrast with the brain, the expression levels of MeCP2 protein in the heart are reportedly higher in embryonic stages than in postnatal heart [41]. Therefore, we investigated the expression levels of *Mecp2* during development in heart, liver and kidney. Our analyses showed that in heart, *Mecp2* expression fluctuated during embryonic stages and was higher than at postnatal day 25 (P25) and P30. In kidney, the expression of *Mecp2* declined after P20 and in liver the expression of *Mecp2* appeared to be induced after birth (P00) but remained unstable up to the age of P30 (Additional file 5: Figure S3a-c).

### *CDKL5*expression in Humans and Mouse

The RefSeq database annotates one TSS for *CDKL5* in mouse and two TSSs in human (Additional file 2: Table S2). Our analyses identified two TSSs within 100 bp of the annotated TSS in mouse and the upstream TSS in human samples (p1@CDKL5/Cdkl5 and p2@CDKL5/Cdkl5, Figures 2c and 2f). In both human and mouse samples, *CDKL5* expression was higher in brain tissues than in primary cells or cell lines (Figure 1e and 1f). The two TSSs of *CDKL5* were co-expressed ubiquitously in human and mouse, however p1@CDKL5 was expressed more than p2@CDKL5 in most tissues in humans suggesting that transcripts arising from p1@CDKL5 may be over-represented in humans (Figure 1e and 1f). In mouse p1@Cdkl5 and p2@Cdkl5 were expressed at similar levels in some brain sub-regions. We tracked the expression of *Cdkl5* in mouse heart, liver and kidney over development from embryonic day 11 to postnatal day 30. These tissues were previously reported to have undetectable levels of *Cdkl5*[22, 23]. In heart, the expression levels of *Cdkl5* fluctuated up to P30 (Additional file 5: Figure S3d p1@Cdkl5 p2@Cdkl5 heart). In liver and kidney, Cdkl5 expression from both TSSs was lower in adult (P25 and P30) than embryonic tissues (Additional file 5: Figure S3e,f). This observation was in contrast to the brain where the expression of *Cdkl5* was generally higher in postnatal brain in both mouse and humans (Additional file 6: Figure S5e,f). In agreement with published data [21] we found restricted expression of *Cdkl5* in mouse astrocytes (maximum 2 TPM, Additional file 3: Figure S1e).

### Developmental profile for the three genes in brain sub regions

The expression levels of *FOXG1*, *MECP2* and *CDKL5* are developmentally regulated in the brain. *FOXG1* expression is reported to be highest during early embryogenesis [2, 42, 43]. CDKL5 is weakly expressed during embryogenesis in the cortex and its expression increases in postnatal stages until P14 after which CDKL5 expression is diminished [21, 24]. MeCP2 protein levels in the brain increase as development proceeds stabilizing around postnatal day 5 [19]. We investigated if the reported developmental expression profile for the three proteins was reflected at the TSS level. For mouse, we investigated the developmental TSS expression of *Mecp2* and *Cdkl5* in the cerebellum (n = 3 at each age), pituitary cortex (n = 1 at each age) and visual cortex (n = 4 at P15 and n = 3 at P30 and P60). Our data reveal that p1@Mecp2 expression fluctuates in embryonic cerebellum samples but is clearly induced after postnatal day 9 (Additional file 7: Figure S4). The expression of p1@Cdkl5 and p2@Cdkl5 in mouse closely resemble the pattern of expression of p1@Mecp2 but at lower levels. These data are in agreement with protein expression levels of Cdkl5 and Mecp2 reported previously in cerebellum by Rusconi et al. [21]. In visual cortex samples, where we investigated a broader time course we found a striking resemblance of expression pattern between p1@Mecp2 and p2@Cdkl5 with both genes showing an increase from P14 to P30 and stabilizing from P30 to P60, while the expression of p1@Cdkl5 remained steady. In contrast, the expression of pA@Foxg1 decreased as visual cortex matured (Additional file 7: Figure S4b). Similarly, in the pituitary gland, we found the expression of *Cdkl5* and *Mecp2* to fluctuate during embryonic stages, while *Foxg1* displayed high expression during embryonic stages with lowest expression in adult.

In broad time-course samples of fetal, neonate and adult human brain sub-regions we found that FOXG1 expression was generally higher in fetal samples (Additional file 6: Figure S5a) while the expression of both promoters of *CDKL5* and p1@MECP2 in fetal samples were lower than their expression level in adults (Additional file 6: Figure S5b,c).

### Comparison between *Mecp2*and Histone H1 expression level in neurons

Skene *et al*. previously showed that in wild-type mouse neurons, the density of MeCP2 protein is one molecule per two nucleosomes - equal to that of Histone H1, which is also one molecule per two nucleosomes [19]. We investigated whether the similarity between Mecp2 and Histone H1 protein density was reflected at the mRNA level in human and mouse neurons. We also compared the expression level of Histone H1 and *Mecp2* TSS in brain sub regions even though Skene *et al.*[19] had not reported a correlation between the levels of the two proteins in whole brain tissue (they reported a correlation only in neuronal nuclei). We extracted the expression for all H1 transcripts and compared their individual and collective levels to p1@MECP2 expression in both human and mouse. The primary cells in mouse included neurons from various brain sites as well as astrocytes and microglia cells. Our data show that the combined as well as individual expression levels of all histone H1 transcripts were much higher than the expression levels of p1@Mecp2 at all ages in all samples (Additional file 8: Figure S6). In raphe neurons, substantia nigra neurons (E14), ventral spinal cord neurons (E14) and hippocampal astrocytes, we found the expression level of a few histone transcripts closer to the expression level of *Mecp2* (Additional file 9: Table S3). Overall, this is interesting, because in order to reconcile this with the findings of Skene *et al.,* substantial changes in either protein production or decay of MeCP2 and/or the Histone 1 proteins must occur to offset the mRNA steady state.

### Intra gene and Inter gene expression correlations between *FOXG1*, *CDKL5*and *MECP2*in brain sub-regions

To investigate the relationship between the expression of all TSSs of each gene, we conducted intra gene correlations and found a high degree of correlation between p1@FOXG1 and p3@*FOXG1* in human samples and between the promoters pA@Foxg1, pB@Foxg1, p1@Foxg1 and p2@Foxg1 in mouse (Table 1, Additional file 10: Table S4, Additional file 11: Figure S7). The two TSSs of *MECP2* were moderately but positively correlated with each other in humans and mouse, while the two TSSs of *Cdkl5* were highly correlated in mouse (correlation coefficient, Spearman’s rank correlation 0.85) confirming our earlier observation of similarities in expression of the two *Cdkl5* promoters in mouse.

**Table 1:** **Spearman Rank Correlations between the key promoters of the three genes in mouse (A) and human (B)**

A: Correlation between promoters in mouse samples						
	pA@Foxg1	pB@Foxg1	p1@Foxg1	p1@Mecp2	p1@Cdkl5	P2@Cdkl5
pA@Foxg1	1.00	0.77	0.76	0.35	0.24	0.29
pB@Foxg1	0.77	1.00	0.83	0.32	0.22	0.26
p1@Foxg1	0.76	0.83	1.00	0.30	0.21	0.24
p1@Mecp2	0.35	0.32	0.30	1.00	0.49	0.56
p1@Cdkl5	0.24	0.22	0.21	0.49	1.00	0.85
P2@Cdkl5	0.29	0.26	0.24	0.56	0.85	1.00
**B: Correlation between promoters in human samples**						
	**P1@FOXG1**	**P2@FOXG1**	**P3@FOXG1**	**p1@MECP2**	**p1@CDKL5**	**P2@CDKL5**
P1@FOXG1	1.00	0.75	0.81	0.06	0.23	0.20
P2@FOXG1	0.75	1.00	0.77	0.08	0.30	0.26
P3@FOXG1	0.81	0.77	1.00	0.06	0.24	0.22
p1@MECP2	0.06	0.08	0.06	1.00	0.23	0.27
p1@CDKL5	0.23	0.30	0.24	0.23	1.00	0.73
P2@CDKL5	0.20	0.26	0.22	0.27	0.73	1.00

See Additional file 9: Table S4 for a complete list.

**Figure 3:**
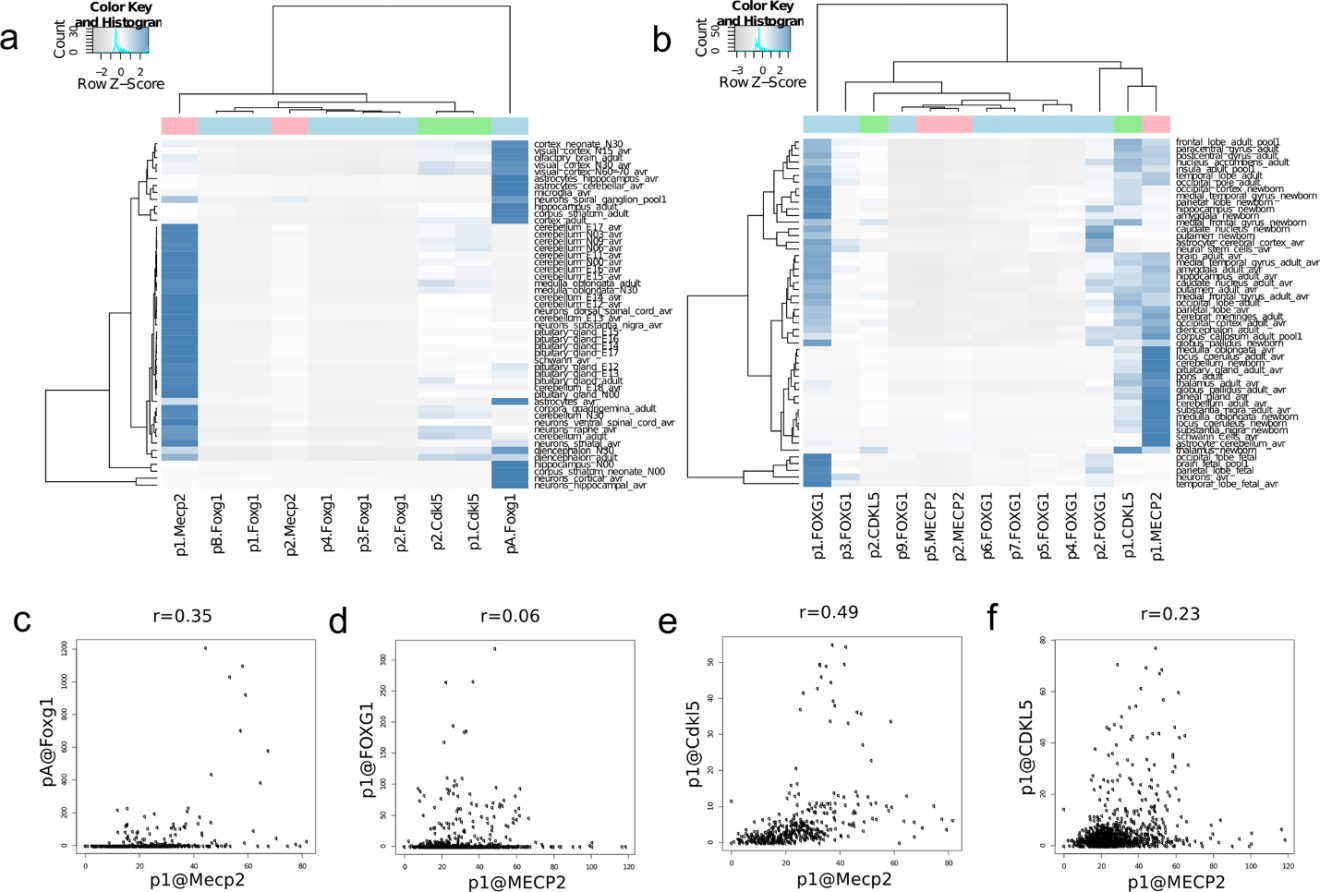
**Expression correlations between the three genes.** Heat maps showing the TPM expression of all promoters in sub-regions of brain and brain related primary cells in mouse **(a)** and humans **(b)**. Expression of *Mecp2* p1 appears to be in contrast with the expression of the main promoter of *Foxg1* in mouse (pA) and in humans (p1). The trees above the heatmaps show clustering according to expression. Plots in panels c to f show correlation between the three genes in mouse **(panels c and e)** and human **(panels d and f)** as labeled. The expected negative correlation based on the heatmap between *MECP2* and *FOXG1* could not be confirmed in either species across all samples **(panels c and d)**. We found positive correlation (23-49%) between *Cdkl5* and *Mecp2* in both species **(panels e and f)**.

Since mutations in the *MECP2*, *FOXG1* and *CDKL5* genes result in overlapping neurological phenotypes, we additionally investigated the inter gene expression correlations of the three genes in the brain. We first generated heatmaps from all brain sub-regions and brain related primary cells (Figure 3a and b) for the three genes. We found that in humans and mouse the expression of p1@FOXG1 and pA@Foxg1 in brain was strikingly discordant with the expression of p1@MECP2 and p1@Mecp2 respectively (Figure 3a, 3b). Next, we investigated the correlation between the highly expressed promoters of the three genes using Spearman’s rank test and Pearson’s correlation coefficients. Based on our earlier heatmap visualization of contrasting expression of *MECP2* and *FOXG1* in brain tissue, we expected to see a negative correlation between the promoters of these genes in both species. Our data generated from all brain tissues, neurons and astrocytes showed that in mouse the correlation between pA@Foxg1 and p1@Mecp2 expression was poor (0.3) and in humans we found negative correlation of −0.1, suggesting slight discordance of expression of the two genes in brain. Thus, our analyses failed to find mathematically significant evidence of contrasting expression between *FOXG1* and *MECP2*. The two promoters of *CDKL5* were also poorly correlated with the F*OXG1* promoter expression in brain, while there was a positive correlation (23-49%) between expression of *MECP2* and *CDKL5* in both species (Figures 3c - 3f, Additional file 11: Figure S7).

### Identification of regulatory regions of the three genes

To identify the regulatory regions associated with the TSS of the three genes in mouse, we extended our TSS co-ordinates by 500bp on either side and intersected them with active histone regulatory marks of H3K27ac and H3K4me3 from ENCODE datasets. Based on current literature [44–47], on chromatin modifications marking active enhancers and promoters, we defined the criteria for active enhancers as those regions carrying the H3K27ac mark and active promoters as those regions carrying the H3K4me3 without complete overlap with the H3K27ac mark.

**Figure 4:**
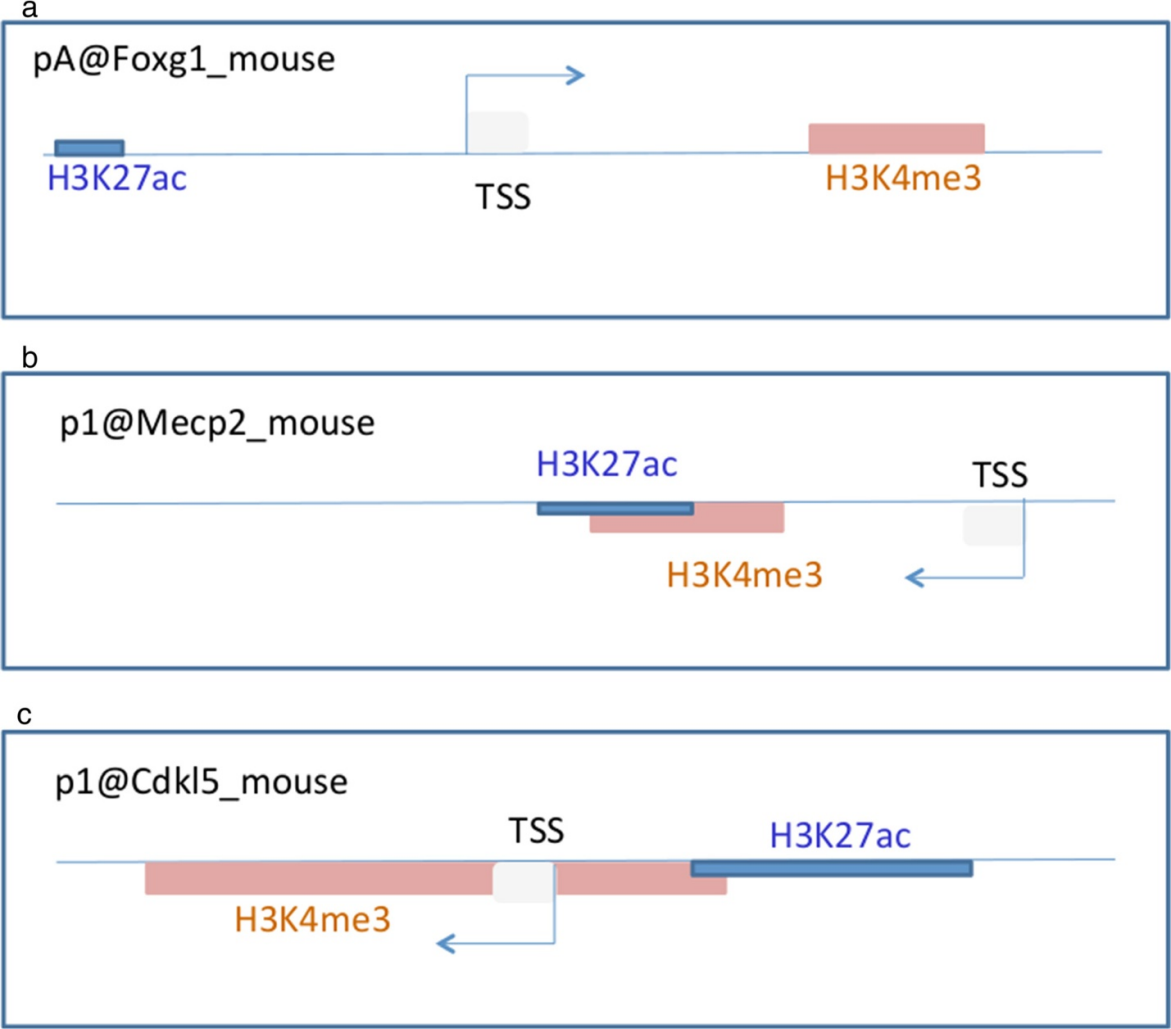
**Mouse gene models derived from FANTOM5 TSS and ENCODE ChIP data.** Gene models for *Foxg1* **(panel a)**, *Mecp2* **(panel b)** and *Cdkl5* **(panel c)** were drawn for the main TSS for each gene and the ENCODE histone ChIP marks for 8 week mouse cortex. For *Foxg1*, the enhancer mark was 1 kb upstream and the promoter mark was 1.1 kb downstream of the TSS. For *Mecp2*, the TSS was upstream of the overlapping promoter and enhancer mark. For *Cdkl5*, the TSS was within the promoter and the enhancer was upstream of the TSS.

Since all three genes were found highly expressed in the cortex, we used ENCODE ChIP tracks for 8 week old cortex for this analysis. Based on our results we derived gene models for the three genes in mouse cortex (Figure 4). Our data revealed that the investigated regulatory marks for mouse *Foxg1*, were distinct and non-overlapping (Additional file 12: Table S5). We found that the main TSSs pA@Foxg1 *and* pB@Foxg1 were located between an enhancer specific histone mark upstream and a promoter specific histone mark downstream (Figure 4a and Additional file 12: Table S5). In contrast, for *Mecp2,* we found the enhancer and promoter specific histone marks to coincide in this tissue (Figure 4b, Additional file 12: Table S5). The *Mecp2* TSSs p1@Mecp2 and p2@Mecp2 were upstream but within 500 bp of the histone specific marks for enhancer and promoter. For *Cdkl5*, we found a partial overlap between enhancer and promoter specific histone marks. The p1@Cdkl5 was located within the promoter specific histone mark while the enhancer specific histone mark was found upstream (Figure 4c, Additional file 12: Table S5). The TSS p2@Cdkl5 was also located within 500 bp of these marks.

As a complementary analysis, we identified human enhancers for the three genes using the database provided in [34] that predicts active enhancers based on the expression of balanced bi-directional low expressed enhancer RNA transcripts. For *FOXG1*, *MECP2* and *CDKL5, we* found 4, 14 and 1 significantly correlated cis-enhancers respectively (Additional file 13: Table S6, Additional file 14: Figure S8). In contrast to the mouse cortex data, the predicted enhancers in human samples were found kilo bases away from each gene suggesting long-range complex regulation of the three genes in humans. For *FOXG1* the most highly correlated enhancer (r = 0.78) was located 7kb upstream of the gene. Many predicted enhancers for *MECP2* had an average correlation of 0.37, the closest enhancer (53kb) had an expression correlation of 0.43, while the highest correlated enhancer (r = 0.55) was over 408kb distant. For *CDKL5*, the only identified active enhancer had a low correlation of 0.2 and was located over 245 kb upstream of the gene (Additional file 13: Table S6). Interestingly, our data revealed that in humans, the only enhancer displaying the expected high correlation with gene expression was for the tissue specific gene *FOXG1*.

### CpG/TATA regulation of the three Rett genes and their promoter shapes

To investigate the regulation of the promoters of the three genes, we analyzed computationally, the presence of CpG islands and TATA boxes in the vicinity of the promoters of the three genes. Intersections of CpG and TATA UCSC bed files, with our extracted list of TSSs, revealed that the three genes had TATA-less promoters in both species. Our data showed both promoters of *MECP2* and *CDKL5* within CpG islands in both species. For *FOXG1*, 4 TSSs in mouse and 3 TSSs in human samples appeared to be regulated by CpG islands (Table 2).

**Table 2:** **List of all transcript initiation sites for the 3 genes in mouse (A) and human (B) samples with their shapes and association with TATA-box and CpG islands**

A Mouse promoters			
Promoter	TATA	CpG	Shape
pA@Foxg1	TATA-less	CpG	Broad
pB@Foxg1	TATA-less	CpG-less	Broad
p1@Foxg1	TATA-less	CpG-less	Sharp
p2@Foxg1	TATA-less	CpG	Broad
p3@Foxg1	TATA-less	CpG	Broad
p4@Foxg1	TATA-less	CpG	Broad
p1@Mecp2	TATA-less	CpG	Broad
p2@Mecp2	TATA-less	CpG	Broad
p1@Cdkl5	TATA-less	CpG	Broad
p2@Cdkl5	TATA-less	CpG	Sharp
**B Human promoters**			
**Promoter**	**TATA**	**CpG**	**Shape**
p1@FOXG1	TATA-less	CpG	Broad
p2@ FOXG1	TATA-less	CpG-less	Sharp
p3@ FOXG1	TATA-less	CpG-less	Sharp
p4@ FOXG1	TATA-less	CpG	Broad
p5@ FOXG1	TATA-less	CpG-less	Broad
p6 @FOXG1	TATA-less	CpG-less	Broad
p7@ FOXG1	TATA-less	CpG	Broad
p9@ FOXG1	TATA-less	CpG-less	Sharp
p1@MECP2	TATA-less	CpG	Broad
p2@ MECP2	TATA-less	CpG	Broad
p5@MECP2	TATA-less	CpG-less	Broad
p1@CDKL5	TATA-less	CpG	Broad
p2@CDKL5	TATA-less	CpG	Sharp

Promoters were listed as TATA-less if a TATA-box was absent 500 bp upstream of the promoter. Similarly in the absence of a CpG island within 500 bp of the TSS, the promoter was classified as CpG-less.

**Figure 5:**
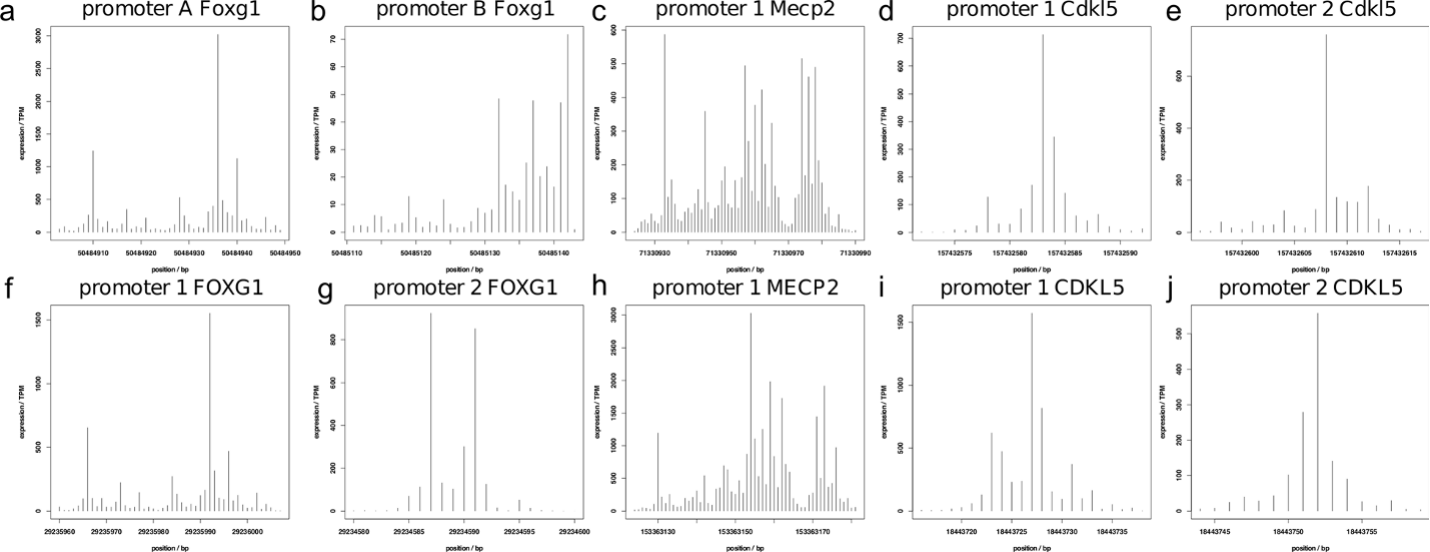
**Shapes of key promoters of the three genes.** Promoter shapes were drawn for the key promoters of the three genes **(panels a to j**, as labeled) based on the location of the first nucleotide in all tissues in mouse **(panels a to e)** and humans **(panels f to j)**. Shape conservation is seen across the two species in all promoters except pB of *Foxg1* in mouse and p2 of *FOXG1* in humans. Despite the closeness in location and high correlation between p1 and p2 of *CDKL5*, we find variation in their shapes suggesting differential regulation in both species.

It is known that promoters regulated by TATA boxes are ‘sharp’ where transcript initiation occurs at a well defined dominant site, no more than 4 consecutive nucleotides long, while promoters regulated by CpG islands are ‘broad’ where multiple start sites can be detected in a broad genomic region [48]. We analyzed the extracted TSSs for sharp or broad shapes by aligning their expression levels across the genomic locus. For our investigation, we defined sharp promoters as those where the majority of the transcripts start from a single dominant TSS or from multiple TSSs within 5 nucleotides, while promoters were classified as broad when they had multiple dominating initiation sites within a defined TSS cluster (maximum 50 bp genomic window). We analyzed in humans and mouse, the 3 main promoters for *FOXG1* and two promoters each for *MECP2* and *CDKL5*. Our analyses revealed that the main promoters for *FOXG1* in both species (p1@FOXG1 and pA@Foxg1) were broad in keeping with the CpG islands in their vicinity (Figure 5a, 5f). The second highest expressed FOXG1 promoters in human and mouse (p2@FOXG1 and pB@Foxg1) appeared to have species-specific shapes and regulation. While p2@FOXG1 in humans was found to be sharp with no TATA-box or CpG island, pB@Foxg1 in mouse was broad and CpG regulated (Figure 5b, 5g). In each species we found for FOXG1, one sharp promoter (p3@FOXG1 and p1@foxg1) devoid of TATA box or CpG island (Additional file 15: Figures S9a,f).

The two main promoters of *MECP2* were broad, in agreement with the CpG islands near their TSS (Figure 5c,h and Additional file 15: Figures S9e,l). In both species, the promoter p1@CDKL5 was broad in shape while p2@CDKL5 was sharp despite the presence of a CpG island in its vicinity (Figure 5d, e ,i and j). A comprehensive list of promoters, their regulation and shapes is shown in Table 2 and Additional file 15: Figure S9.

### Transcriptional regulation of the three genes

To identify transcription factors binding to the three genes in both species, we analyzed the genomic sequence within 500 bp from the promoters of the three genes using the SwissRegulon database of sequence motifs associated with transcription factors [49] (see Methods for details). We found a putative binding site with a posterior probability greater than 0.7 in the human *FOXG1*, *MECP2* and *CDKL5* promoter regions for 23, 11 and 14 TFs respectively (Additional file 16: Table S7A). Of these, binding sites for the three transcription factors RREB1, FOXP1 and NFY were found in all three genes, suggesting that the three genes implicated in Rett syndrome may be regulated by the same TFs in humans. We then summed the posterior probabilities over each promoter region to estimate the number of binding sites for each transcription factor and evaluated its statistical significance (Table 3). The data reveal that in human, the sequence around the main promoter of *FOXG1* in human was significantly enriched in binding sites for the RREB1 (*p* = 0.01), FOXP1 (*p* = 0.03), and NFY (*p* = 0.01) transcription factors. NFY was also predicted to regulate *MECP2* (*p* = 0.01) and possibly *CDKL5* (*p* = 0.09). Similar analyses in the mouse genome revealed motifs for 21, 5 and 3 TFs within 500 bp of the *Foxg1*, *Mecp2* and the *Cdkl5* promoters respectively (Additional file 15: Table S7B). In mouse although all TFs with binding sites in the *Mecp2* and *Cdkl5* promoter regions also appeared to have binding sites in the *Foxg1* promoter region, only 2 TFs (Sp1 and NFY) were common to all three genes. Calculating the statistical significance of the estimated number of binding sites revealed that in mouse for all three genes the promoter regions were enriched for motifs associated with transcription factor NFY, as well as Sp1 (Table 3).

**Table 3:** **Transcription factor binding sites analyses at the promoters of the three genes**

TF	p1@FOXG1	p2@FOXG1	p1@MECP2	p2@MECP2	p1@CDKL5	p2@CDKL5
RREB1	1.5 (*p =* 0.01)*	0.0 (*p =* 1.0)	0.0 (*p =* 1.0)	0.0 (*p =* 1.0)	0.7 (*p =* 0.08)	0.7 (*p =* 0.08)
FOXP1	1.3 (*p =* 0.03)*	0.7 (*p =* 0.1)	0.0 (*p =* 1.0)	0.0 (*p =* 1.0)	0.0 (*p =* 1.0)	0.0 (*p =* 1.0)
NFY	1.9 (*p =* 0.01)*	0.0 (*p =* 1.0)	1.3 (*p =* 0.02)*	1.9 (*p =* 0.01)*	0.9 (*p =* 0.09)	0.9 (*p =* 0.09)
**TF**	**pA@Foxg1**	**pB@Foxg1**	**p1@Mecp2**	**p2@Mecp2**	**p1@Cdkl5**	**p2@Cdkl5**
RREB1	0.0 (*p =* 1.0)	0.9 (*p =* 0.06)	0.0 (*p =* 1.0)	0.34 (*p =* 0.6)	0.0 (*p =* 1.0)	0.0 (*p =* 1.0)
FOXP1	0.0 (*p =* 1.0)	0.0 (*p =* 1.0)	0.0 (*p =* 1.0)	0.2 (*p =* 0.7)	0.0 (*p =* 1.0)	0.0 (*p =* 1.0)
NFY	1.9 (*p =* 0.007)*	1.9 (*p =* 0.007)*	1.3 (*p =* 0.02)*	1.3 (*p =* 0.02)*	1.0 (*p =* 0.03)*	1.0 (*p =* 0.03)*
SP1	1.7 (*p =* 0.04)*	1.4 (*p =* 0.06)	2.5 (*p =* 0.01)*	2.5 (*p =* 0.01)*	2.6 (*p =* 0.01)*	2.6 (*p =* 0.01)*

*Denotes significant values.

## Discussion

Our analyses of the FANTOM5 CAGE data reveal multiple sites for transcript initiation and identify the predominantly used TSSs of the three genes implicated in RTT. Mutation testing for RTT is currently performed solely on known coding exons, even though it has been suggested that the non-coding regulatory regions may play a role in the pathogenesis of RTT [50, 51]. Our data show that the highly used TSSs lie upstream of currently annotated start sites and we propose that these regions be included in testing to ensure accurate representation of genes in diagnosis.

In our investigation we found the expression of *FOXG1* strikingly in contrast with the expression of *MECP2* in the brain, but we could not get firm negative correlation for this observation of discordance in expression. This discrepancy may be due to the high expression level of *FOXG1* transcripts and the variable but comparatively low-level expression of *MECP2* mRNA in the brain. Alternately, our visual observation may have resulted from the fact that some brain regions in mouse (cerebellum and medulla oblongata) and humans (locus coeruleus, pineal gland, cerebellum, medulla oblongata and substantia nigra) are clearly devoid of *FOXG1* expression at any developmental stage. We further confirmed our observation of the absence of *Foxg1* expression in mouse cerebellum through analyses of chromatin signatures from mouse ENCODE. Our investigation revealed enrichment of H3K27me3 in the *Foxg1* genomic region, suggesting PRC2 mediated silencing of *Foxg1* in the cerebellum. Although H3K27me3 has also been reported to be present at transcriptionally active or poised loci [52], the absence of active chromatin marks in the *Foxg1* promoter region in cerebellum but not in the cortex, strongly suggest specific repression of *Foxg1* in the cerebellum. A similar examination in liver also revealed H3K27me3 enrichment at the *Foxg1* promoter region (Additional file 4: Figure S2). It is tempting to propose PRC2 mediated silencing as a universal mechanism to restrict *Foxg1* expression to brain. It is known that PRC2 mediated silencing is facilitated through long ncRNAs [40], but our screening did not reveal potential regulatory long ncRNAs in the vicinity of *Foxg1* suggesting such regulation might be mediated by ncRNAs located outside our window of investigation. It would be interesting to identify the long ncRNAs involved in *Foxg1* silencing and investigate their contribution to the disease phenotype.

Despite the known discrepancy in mRNA and protein levels of *MECP2*[18, 53]), we found that similar to MeCP2 protein [54], *MECP2* mRNA expression was *low* in embryonic stages and high in adult stages in most brain regions except the cerebellum, where its expression was comparatively high in embryonic tissues as well. We also examined the relation between *Histone H1* and *MECP2* at the mRNA level. Our data show that in each brain related sample, Histone H1 transcript expression is 10–1000 fold greater than MECP2 transcript expression. Therefore, for these gene transcripts to produce equal amounts of protein, as suggested in Skene et al., massive up-regulation of protein translation is required from MECP2 transcripts or massive down-regulation of protein translation is needed from Histone transcripts. Thus our data point to another layer of regulatory control between transcription and translation to equalize the protein output from low expressed MECP2 transcripts and abundantly expressed Histone H1 transcripts. The presence of inverted SINE elements in the vicinity of promoters have been reported to up-regulate protein translation [55] but we did not find a similar configuration of SINE near the *MECP2* promoter.

The *MECP2* gene gives rise to two mRNA isoforms with same transcription start site [56, 57] and despite the fact that our analyses revealed two TSSs for *MECP2* in humans and mouse in all tissues, we could not allocate two distinct start sites for the two isoforms of *MECP2.* Based on our data*,* we were unable to conclude whether p2@MECP2/Mecp2 represented an independent poorly expressed protein coding isoform, a shorter non-coding regulatory ncRNA transcript arising from the vicinity of the main promoter p1@MECP2/Mecp2 or a tissue specific enhancer RNA (eRNA) [54] for *MECP2*. Almost 25% of all enhancers are expected to transcribe short bi-directional capped transcripts called e-RNAs [54]. Our observations of stable low expression level of p2@MECP2/Mecp2 sometimes below 5 TPM irrespective of the expression level of p1@MECP2/Mecp2, its poor correlation with p1@MECP2/Mecp2 expression and the absence bi-directional transcripts at p2@*MECP2*/*Mecp2,* do not support its identification as an e-RNA for *MECP2*. The two TSSs for *CDKL5* are highly correlated with each other in mouse as well as humans. Based on their similar expression levels and distinct promoter shapes, we propose that they represent two independently regulated transcripts despite their proximity.

The comparison between corresponding promoters in human and mouse samples, including the novel promoter p1@FOXG1 in human and pA@Foxg1 in mouse, revealed remarkably similar shapes, suggesting evolutionary conservation in their regulation. The only exceptions were the human p2@FOXG1 and pB@Foxg1 mouse, which due to their distinctive promoter shapes appear to be regulated in a species-specific manner.

The recently released ENCODE Histone ChIP seq data [39], allowed us to distinguish, among our identified TSS, active enhancers from active promoters [44, 47] in mouse. Despite the presence of enhancer specific histone mark of H3K27ac, we could not find evidence of low-level antisense transcripts at the enhancer marks in mouse suggesting that enhancers at close range that do not generate e-RNAs may regulate the three genes in mouse. For human samples the histone ChIP data were not available, but we found correlated e-RNAs at distal locations from the TSSs. Our data suggest that in humans the three genes may be regulated by e-RNA producing enhancers at long range. It is unclear at this stage whether this discrepancy reflects true species-specific differences or if it reflects differences in data analyses (Histone marks with no evidence of e-RNAs in mouse vs e-RNAs alone in humans). Further experimental validation is required to confirm whether these regions identified in our study play a regulatory role in the expression of the respective genes.

Almost 20% patients of atypical RTT do not have mutations in the three genes. We conducted genome wide TFBS analyses with the aim to discover the common transcription factors likely to regulate the three genes and thus identify shared pathways upstream. Mutations or functional impairment of such common TFs may affect the expression of the three genes, which may result in disease phenotype. Our data predict that TFs NFY and SP1 are likely to regulate *FOXG1* and *MECP2* but not *CDKL5* in humans and NFY is likely to regulate all three genes in mouse. Further investigation will be needed to experimentally verify these findings nevertheless, it will be of interest to study the expression level and presence of mutations in the common TFs in mutation negative RTT patients.

Our investigations failed to demonstrate brain specific promoter usage or particularly high levels of expression of *MECP2* in brain or neurons, which could have explained the predominantly neurological phenotype seen in patients with mutations in this ubiquitously expressed gene.

## Conclusion

Our comprehensive analyses of data from the FANTOM5 project reveal novel insights into the common and distinct genomic features of the three genes, which are related not only by disease phenotype, but also in their regulation in a species-specific manner.

## Electronic supplementary material

Additional file 1: Table S1: List of all tissues, cells and cell lines with the TPM expression of the FANTOM5 defined transcription start sites of the three genes shown per sample in sheets 1 and 2, and averaged TPM expression across replicates shown in sheets 3 and 4. (XLS 24 KB)

Additional file 2: Table S2: List of RefSeq and FANTOM5 detected transcription start sites in human and mouse. (XLS 24 KB)

Additional file 3: Figure S1: Top 15 samples in expression for each of the three genes. Panels a, c and e represent mouse, while panels b,d and f are human samples showing promoter expression in TPM, on X-axis, in various tissues, as labeled on Y-axis. For each gene, the samples with the highest expression of the main promoter (p1@FOXG1, p1@MECP2 and p1@CDKL5 in human and pA@Foxg1, p1@Mecp2 and p1@Cdkl5 in mouse) are shown. The expression of the other key promoters in these samples is also shown (p2@FOXG1, p3@FOXG1, p2@MECP2 and p2@CDKL5 in human and pB@Foxg1, p2@Mecp2 and p2@Cdkl5 in mouse). (PDF 21 KB)

Additional file 4: Figure S2: Silencing of Foxg1 in mouse. UCSC Browser image of the genomic locus for Foxg1 showing ENCODE tracks for DNAse-I hypersensitive sites, active enhancer specific histone mark (H3K27ac), active promoter specific histone mark (H3K4me3) and PRC2 mediated repressor mark (H3K27me3) in mouse cerebellum, cerebrum, whole brain and liver as labeled. Cerebellum samples lack the DNAse-I hypersensitive sites visible in cerebrum and whole brain samples. Cerebellum samples also lack the active promoter mark H3K4me3 seen in cortex, but contain PRC2 repressive histone mark H3K27me3 not seen in cortex at the locus. (PDF 36 KB)

Additional file 5: Figure S3: Expression levels of Mecp2 and Cdkl5 during development in heart kidney and liver. The line plots show the fluctuations in expression for the two promoters for Mecp2 and Cdkl5 in heart, (a and d), kidney (b and e) and liver (c and f) in mouse. (PDF 46 KB)

Additional file 6: Figure S5: Developmental profile for the 3 genes in human brain. Human *FOXG1* (a), *MECP2* (b) and *CDKL5* (c) expression in TPM across a set of adult, newborn and fetal brain regions is shown as labeled. *FOXG1* shows the highest overall expression as well as having higher expression in fetal than in adult samples as opposed to the expression of *MECP2* and *CDKL5* in the same samples. (PDF 39 KB)

Additional file 7: Figure S4: Expression profile of the three genes in mouse in developing brain tissues. Line plots showing expression of selected promoters of *Foxg1*, *Mecp2* and *Cdkl5* during development in mouse cerebellum (panel a), mouse visual cortex (panel b) and mouse pituitary gland (panel c). Refer main text for details. (PDF 41 KB)

Additional file 8: Figure S6: Comparison of mRNA levels of Histone H1 and *MECP2*. Bar charts showing TPM expression of the key promoter of *MECP2* and collective total expression of Histone H1 TSSs in brain related cells (panels a and c) and tissues (panels b and d) in mouse (panels a and b) and humans (panels c and d). Histone expression levels appear to be over 100 fold higher than *MECP2* in brain related cells suggesting a massive up-regulation of MeCP2 at the level of protein translation. (PDF 39 KB)

Additional file 9: Table S3: Comparison of the expression of the Histone H1 genes promoters and MECP2 in both human and mouse. (XLS 134 KB)

Additional file 10: Table S4: Pearson and Spearman correlations for all TSSs in human and mouse. (XLS 20 KB)

Additional file 11: Figure S7: Intra and inter gene expression correlations between the three genes. Expression correlation plots for all other promoter combinations not present in Figure 3. Plots a-g are mouse promoters, while plots h-n are human promoters as labeled. (PDF 495 KB)

Additional file 12: Table S5: Location of enhancer and promoter specific Histone marks in relation to TSSs in mouse. (XLS 20 KB)

Additional file 13: Table S6: Locations and correlations of human enhancers to the three Rett genes. (XLS 26 KB)

Additional file 14: Figure S8: Locations of active enhancers correlated to the three genes in human samples. UCSC snapshot showing the positions of all eRNA producing human enhancers that are correlated to the expression of the three genes: FOXG1 (a), MECP2 (b) and CDKL5 (c). (PDF 58 KB)

Additional file 15: Figure S9: Promoter shapes for all the other promoters. The shapes of all the individual promoters in mouse (a-e) and human (f-m) are shown as labeled. The shapes are drawn from the first nucleotide of the first mapped CAGE tag to the first nucleotide of the last mapped CAGE tag, the y-axis shows the counts in TPM for each position. (PDF 41 KB)

Additional file 16: Table S7: List of transcription factors with high binding probability of 0.7 and above to the promoters of the three genes in mouse (A) and human (B) genome. Transcription factors common to the three genes are shown in red. (DOC 48 KB)
