# Supplementary material for: CAGE-defined promoter regions of the genes implicated in Rett Syndrome
Source: BMC Genomics. 2014 Dec 24;15(1):1177. doi: 10.1186/1471-2164-15-1177 (PMC4522966; doi:10.1186/1471-2164-15-1177)
Supplement: Supplementary file 3 — Additional file 3: Figure S1: Top 15 samples in expression for each of the three genes. Panels a, c and e represent mouse, while panels b,d and f are human samples showing promoter expression in TPM, on X-axis, in various tissues, as labeled on Y-axis. For each gene, the samples with the highest expression of the main promoter (p1@FOXG1, p1@MECP2 and p1@CDKL5 in human and pA@Foxg1, p1@Mecp2 and p1@Cdkl5 in mouse) are shown. The expression of the other key promoters in these samples is also shown (p2@FOXG1, p3@FOXG1, p2@MECP2 and p2@CDKL5 in human and pB@Foxg1, p2@Mecp2 and p2@Cdkl5 in mouse). (PDF 21 KB) [file 12864_2013_7082_MOESM3_ESM.pdf]

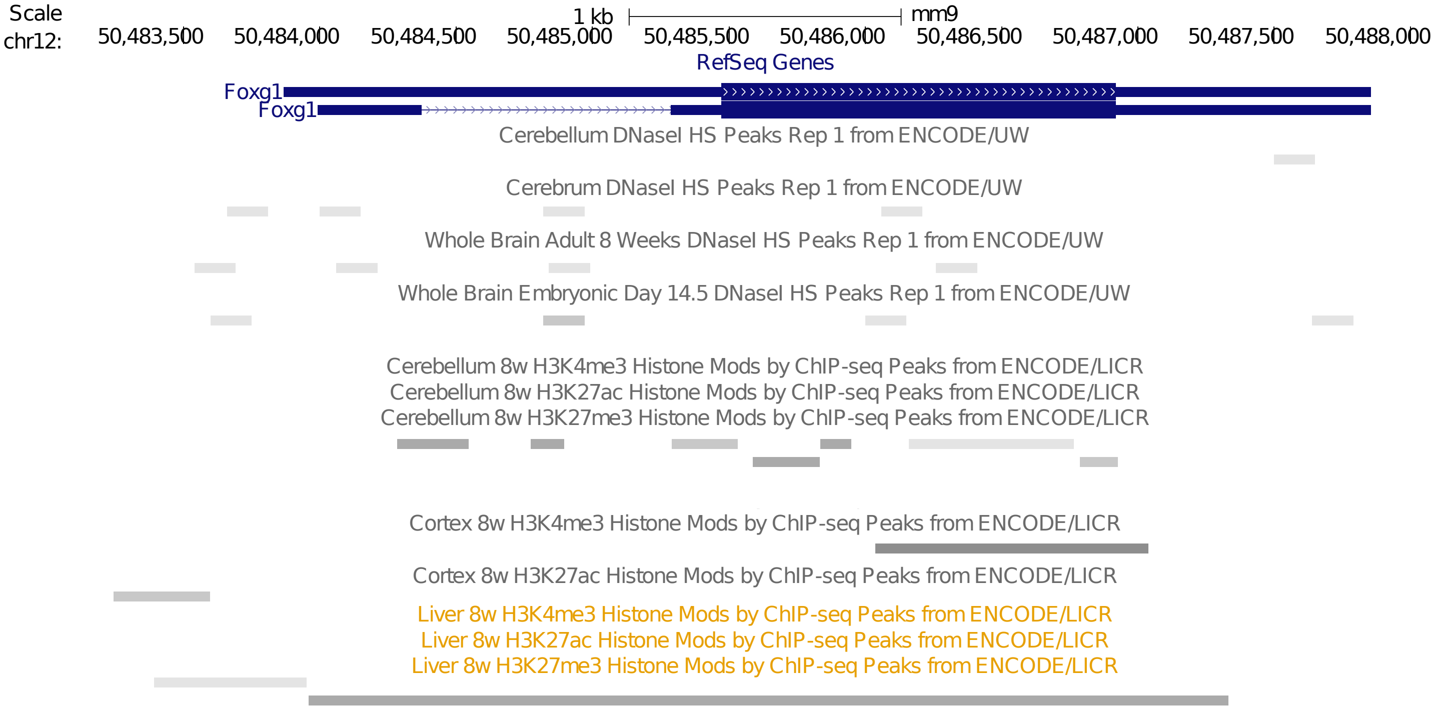
Scale
chr12:
1 kb
mm9
50,483,500
50,484,000
50,484,500
50,485,000
50,485,500
50,486,000
50,486,500
50,487,000
50,487,500
50,488,000
RefSeq Genes
Foxg1
Foxg1
Cerebellum DNaseI HS Peaks Rep 1 from ENCODE/UW
Cerebrum DNaseI HS Peaks Rep 1 from ENCODE/UW
Whole Brain Adult 8 Weeks DNaseI HS Peaks Rep 1 from ENCODE/UW
Whole Brain Embryonic Day 14.5 DNaseI HS Peaks Rep 1 from ENCODE/UW
Cerebellum 8w H3K4me3 Histone Mods by ChIP-seq Peaks from ENCODE/LICR
Cerebellum 8w H3K27ac Histone Mods by ChIP-seq Peaks from ENCODE/LICR
Cerebellum 8w H3K27me3 Histone Mods by ChIP-seq Peaks from ENCODE/LICR
Cortex 8w H3K4me3 Histone Mods by ChIP-seq Peaks from ENCODE/LICR
Cortex 8w H3K27ac Histone Mods by ChIP-seq Peaks from ENCODE/LICR
Liver 8w H3K4me3 Histone Mods by ChIP-seq Peaks from ENCODE/LICR
Liver 8w H3K27ac Histone Mods by ChIP-seq Peaks from ENCODE/LICR
Liver 8w H3K27me3 Histone Mods by ChIP-seq Peaks from ENCODE/LICR
