# Supplementary figures and images for: CAGE-defined promoter regions of the genes implicated in Rett Syndrome
Source: BMC Genomics. 2014 Dec 24;15(1):1177. doi: 10.1186/1471-2164-15-1177 (PMC4522966; doi:10.1186/1471-2164-15-1177)

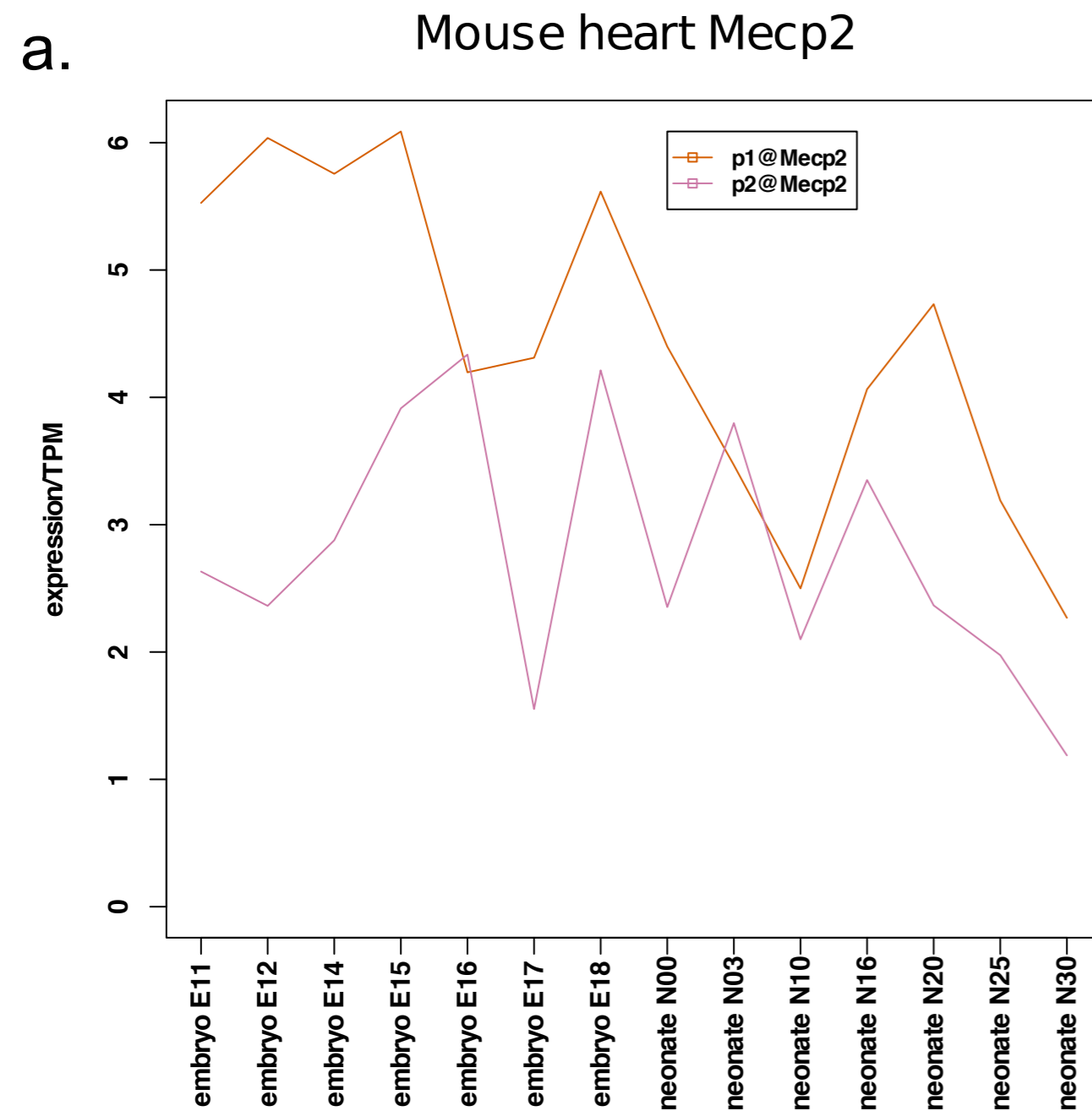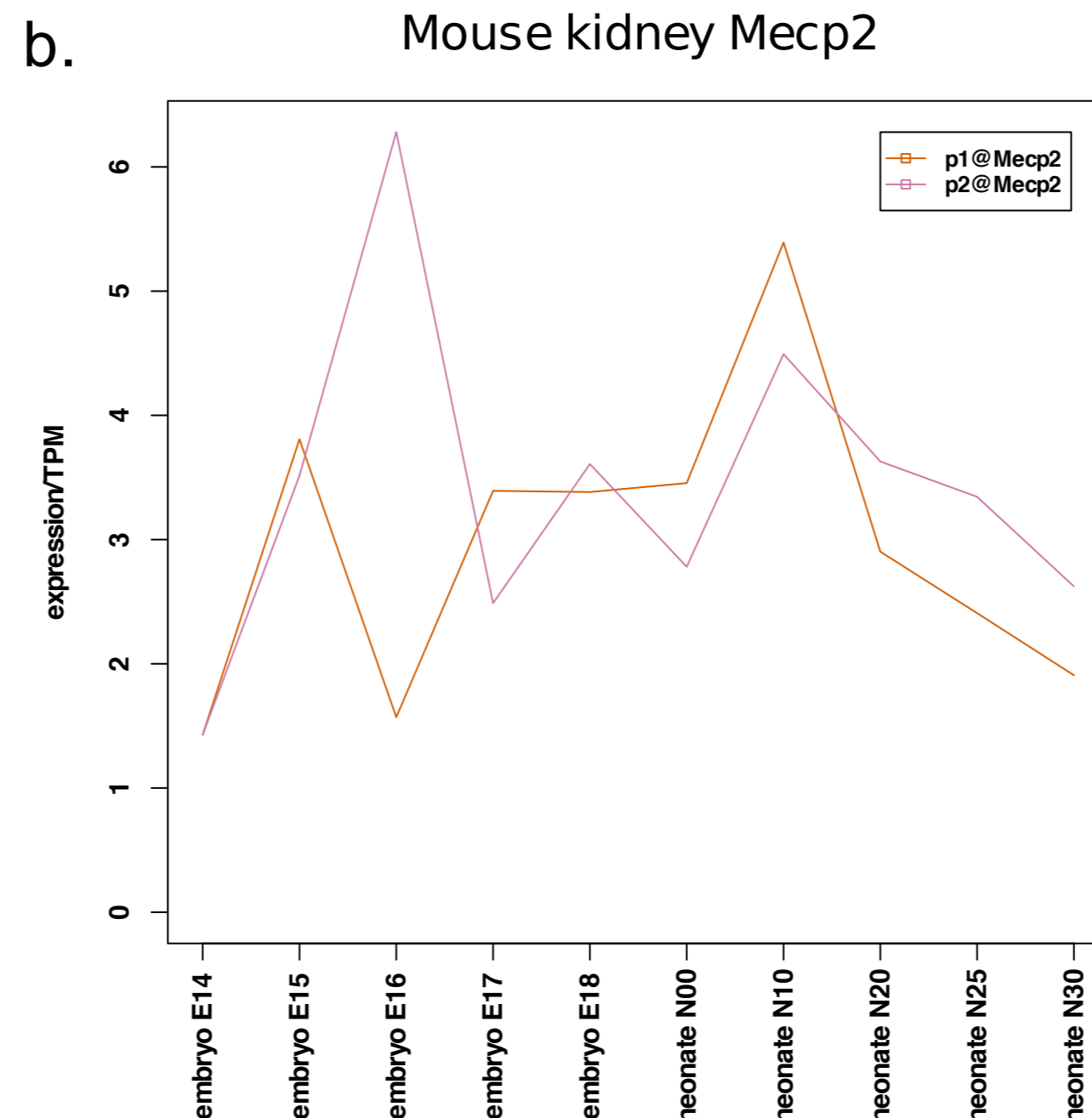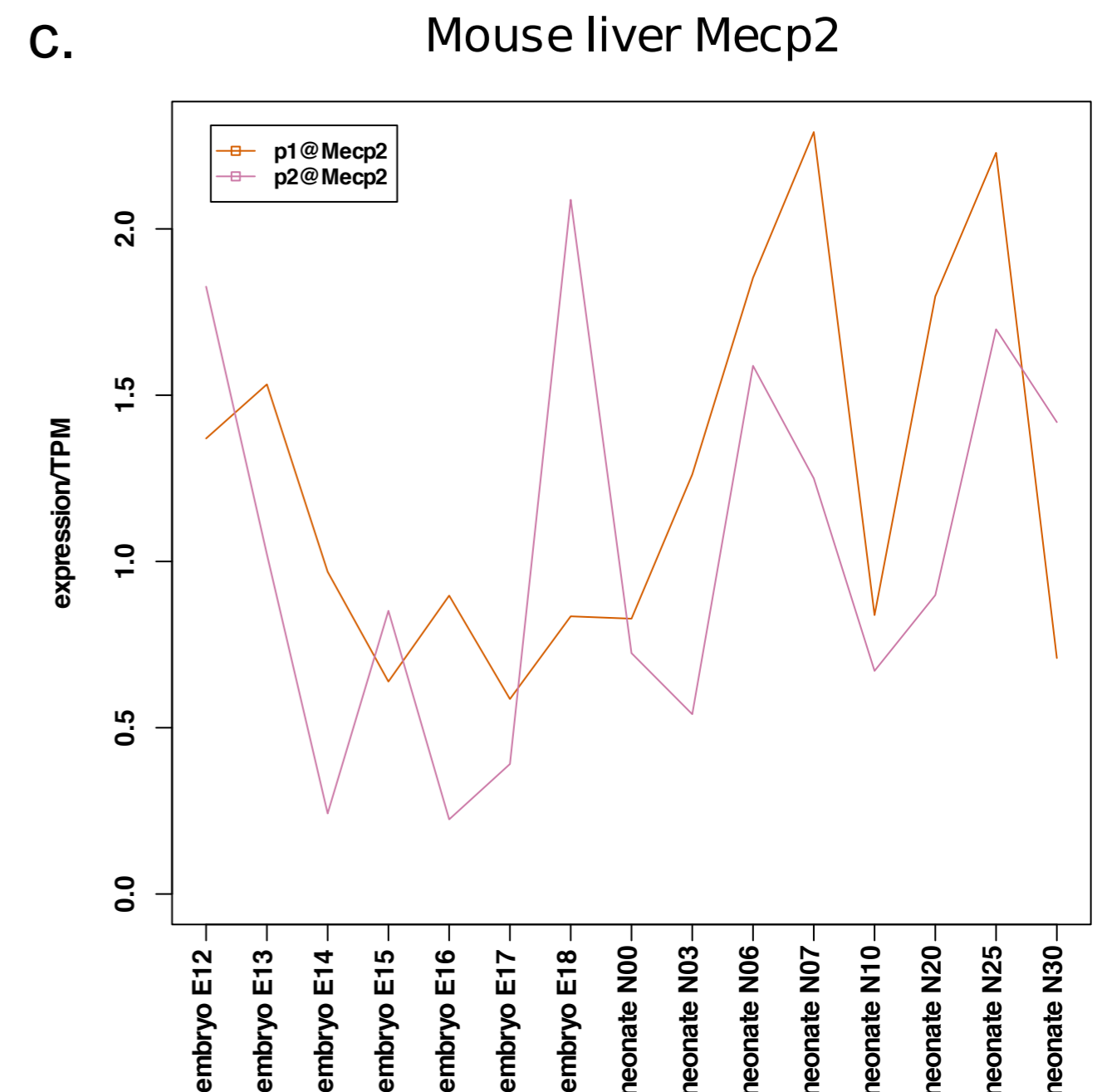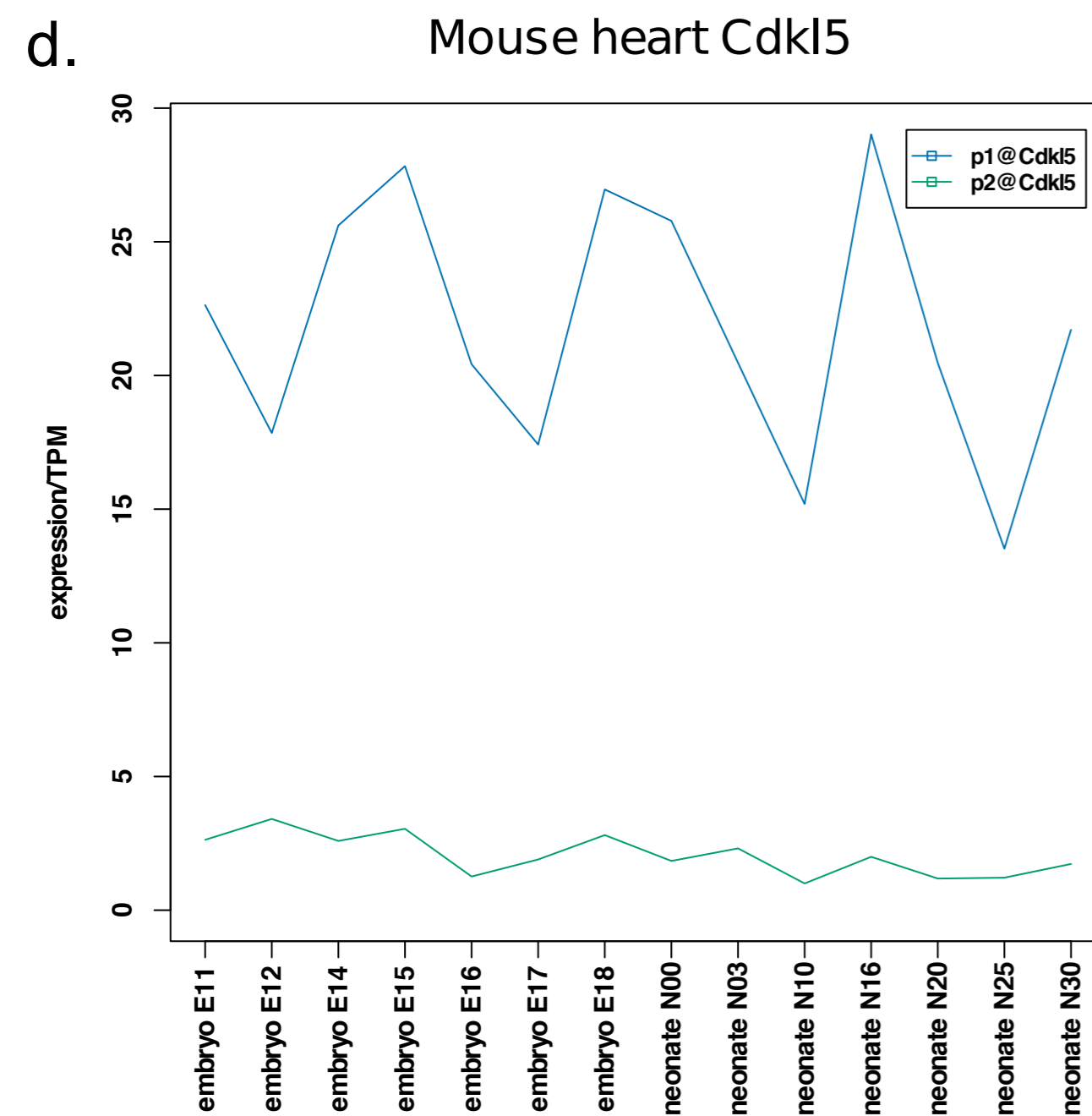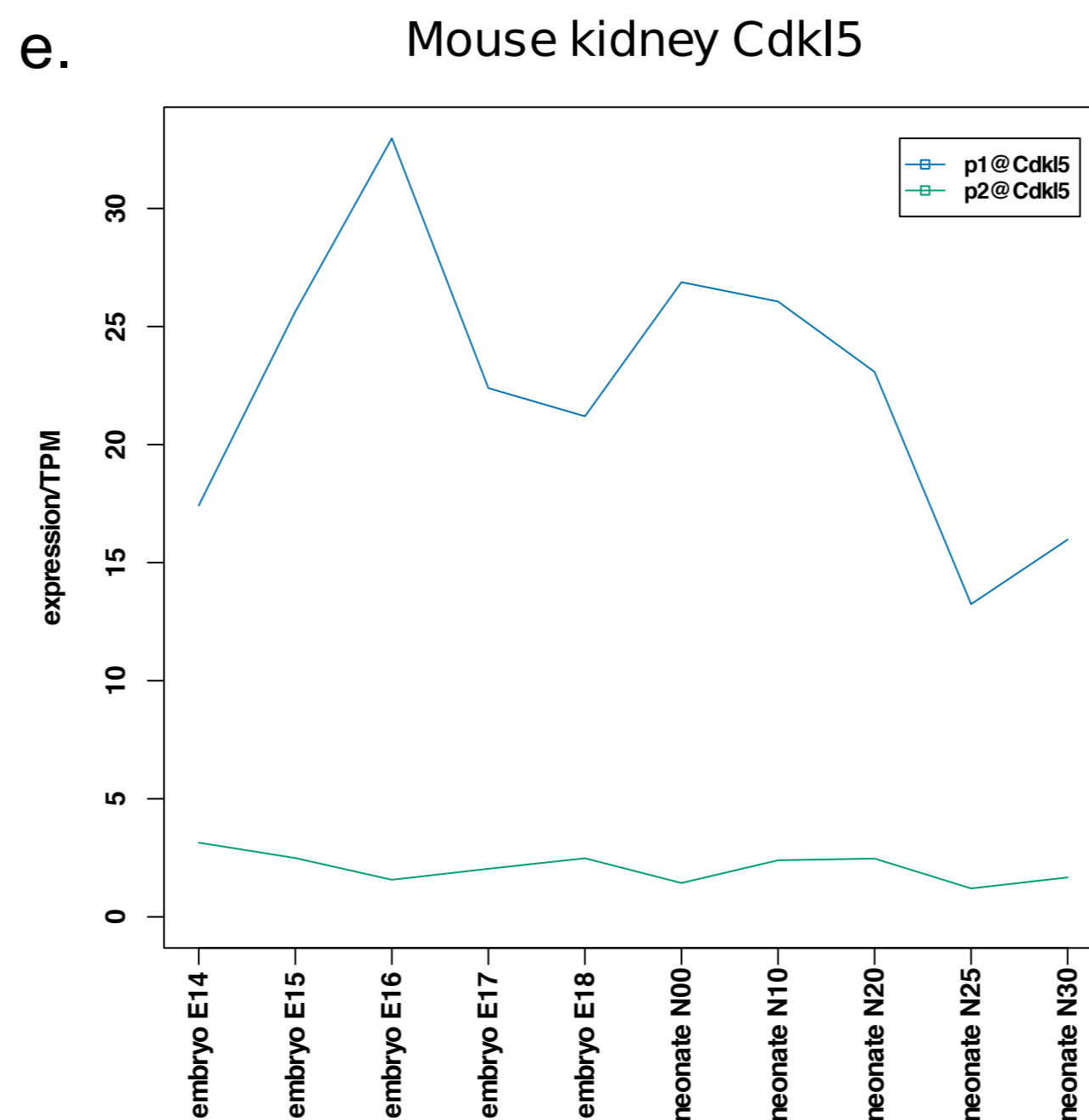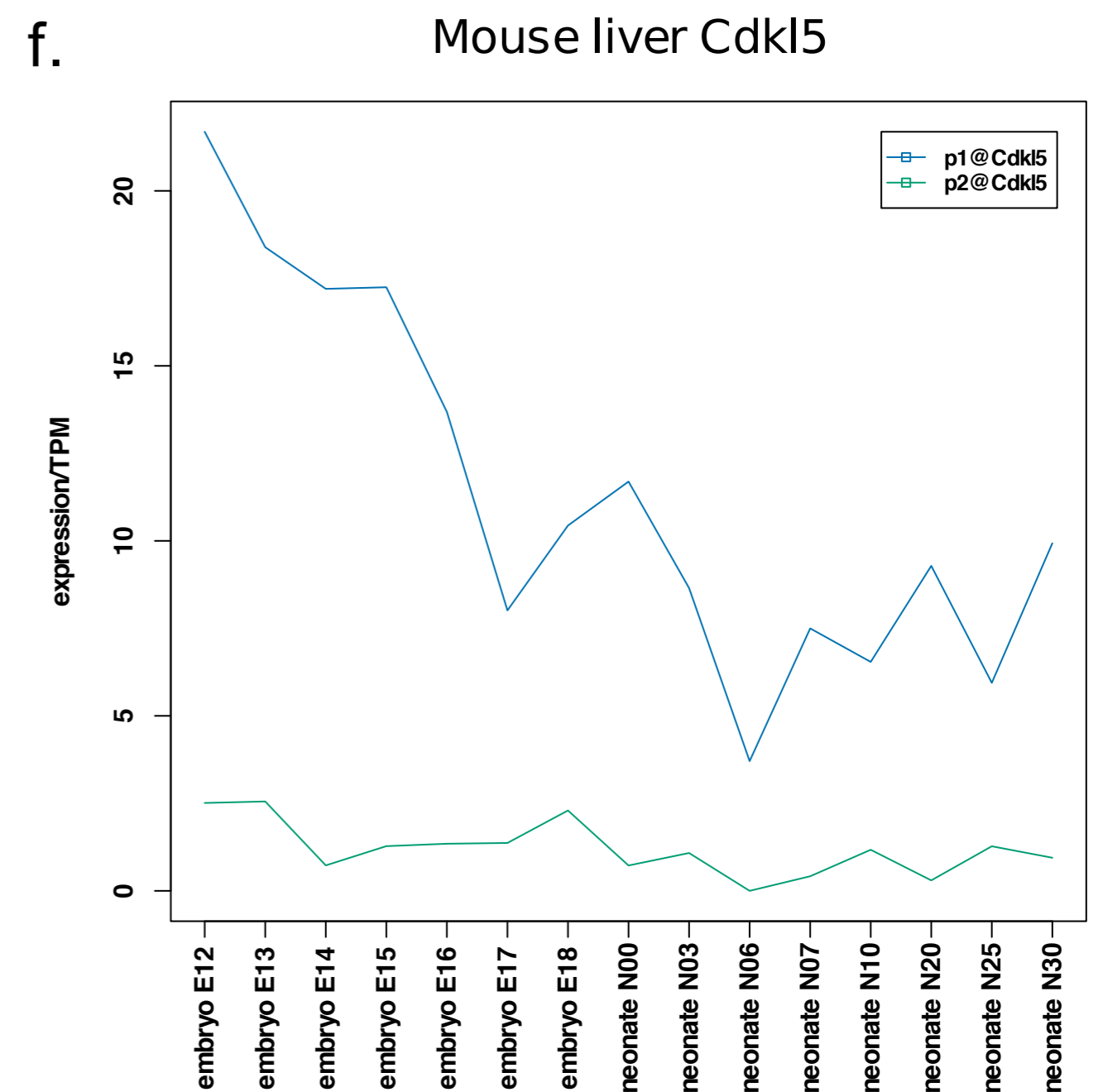

Supplement: Supplementary file 4 — Additional file 4: Figure S2: Silencing of Foxg1 in mouse. UCSC Browser image of the genomic locus for Foxg1 showing ENCODE tracks for DNAse-I hypersensitive sites, active enhancer specific histone mark (H3K27ac), active promoter specific histone mark (H3K4me3) and PRC2 mediated repressor mark (H3K27me3) in mouse cerebellum, cerebrum, whole brain and liver as labeled. Cerebellum samples lack the DNAse-I hypersensitive sites visible in cerebrum and whole brain samples. Cerebellum samples also lack the active promoter mark H3K4me3 seen in cortex, but contain PRC2 repressive histone mark H3K27me3 not seen in cortex at the locus. (PDF 36 KB) [file 12864_2013_7082_MOESM4_ESM.pdf]

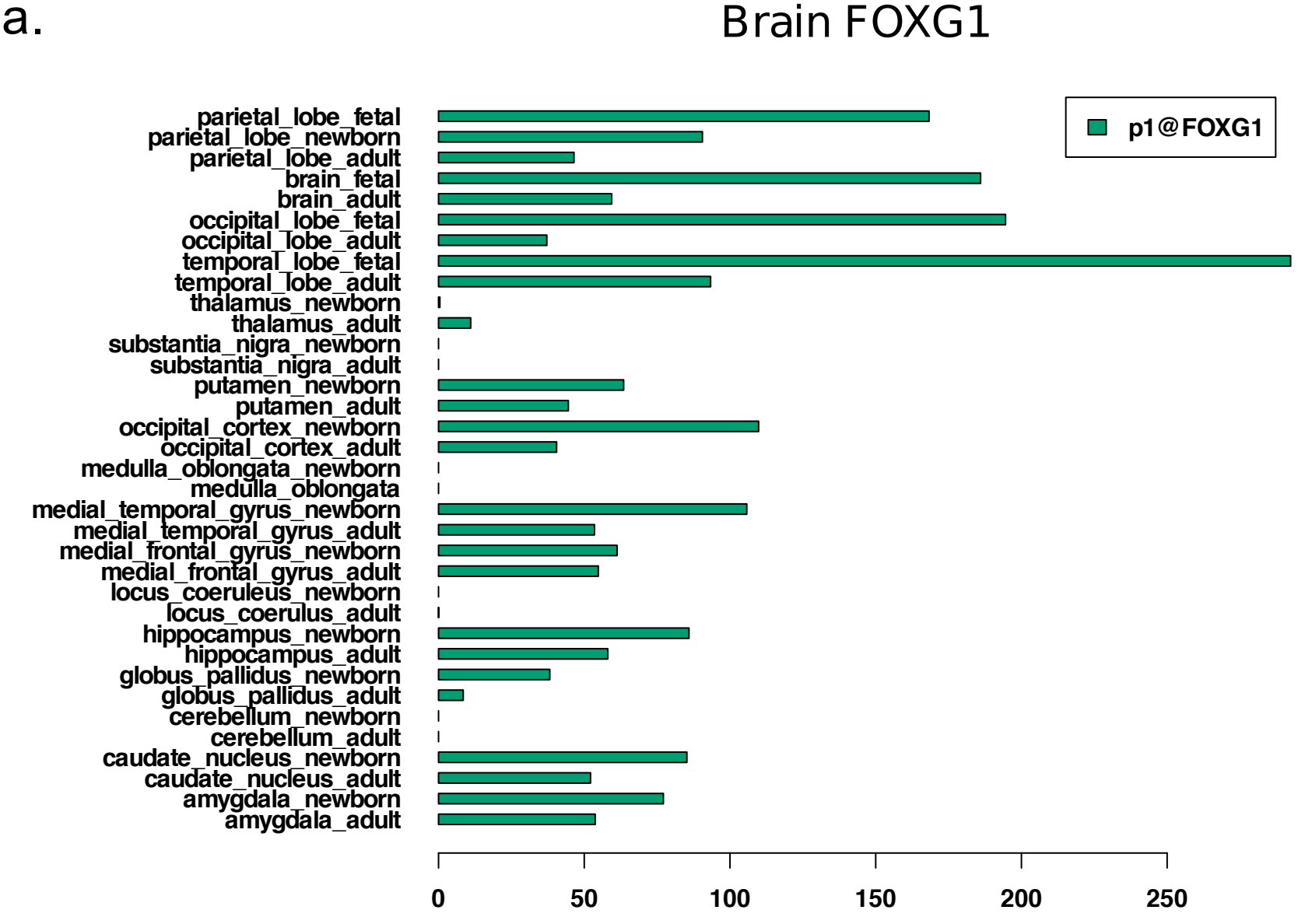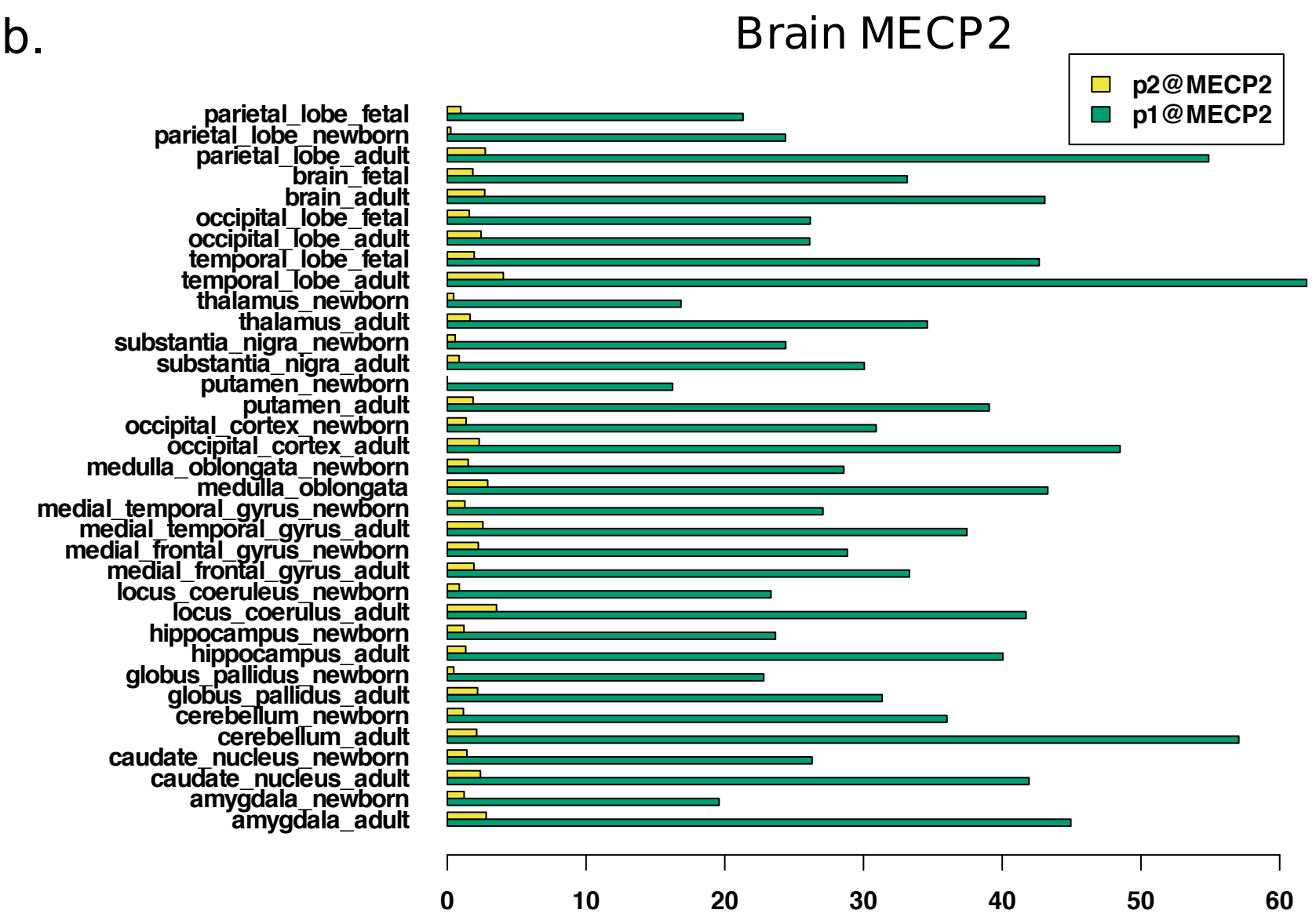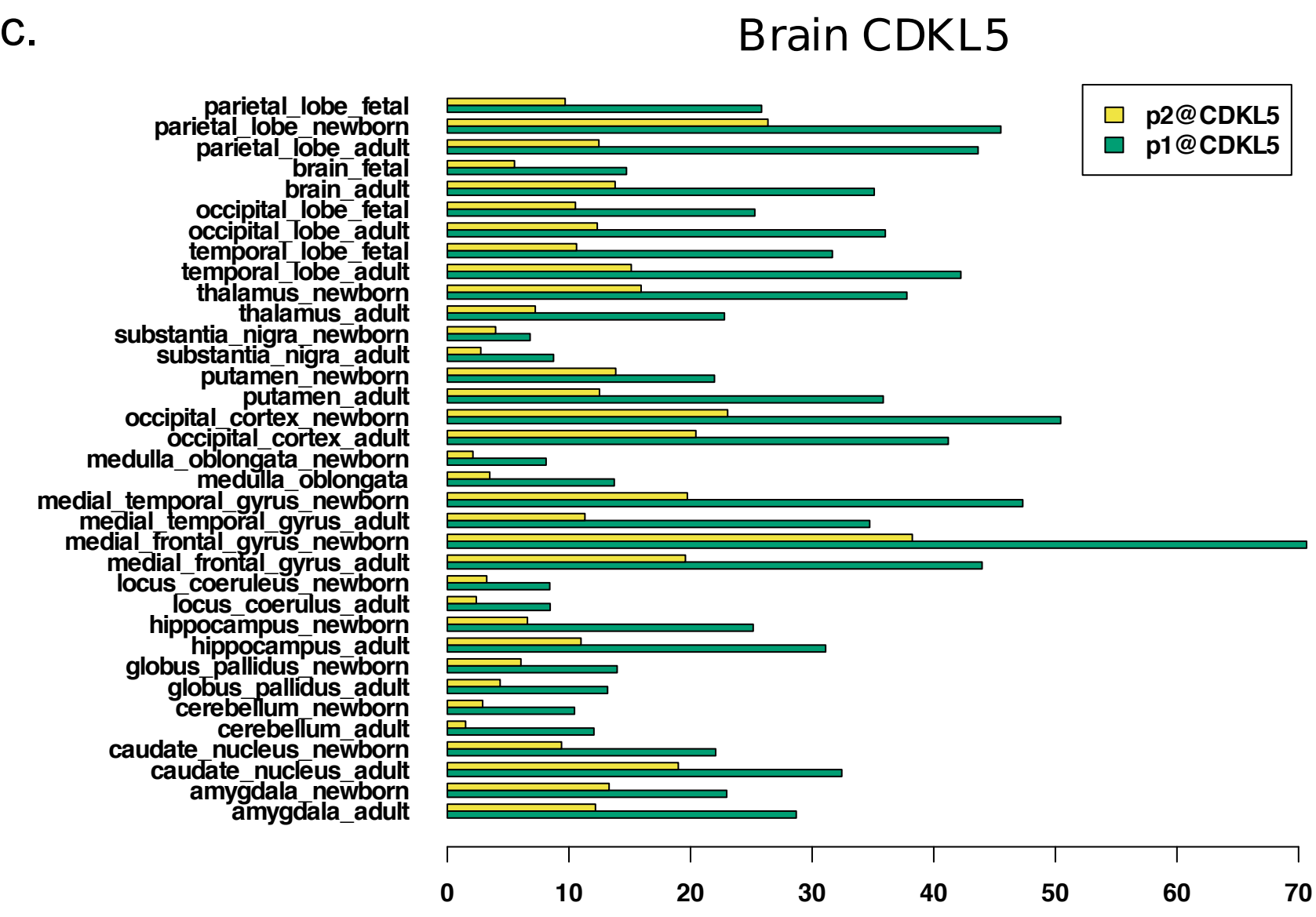

Supplement: Supplementary file 5 — Additional file 5: Figure S3: Expression levels of Mecp2 and Cdkl5 during development in heart kidney and liver. The line plots show the fluctuations in expression for the two promoters for Mecp2 and Cdkl5 in heart, (a and d), kidney (b and e) and liver (c and f) in mouse. (PDF 46 KB) [file 12864_2013_7082_MOESM5_ESM.pdf]

a.

## Mouse cells

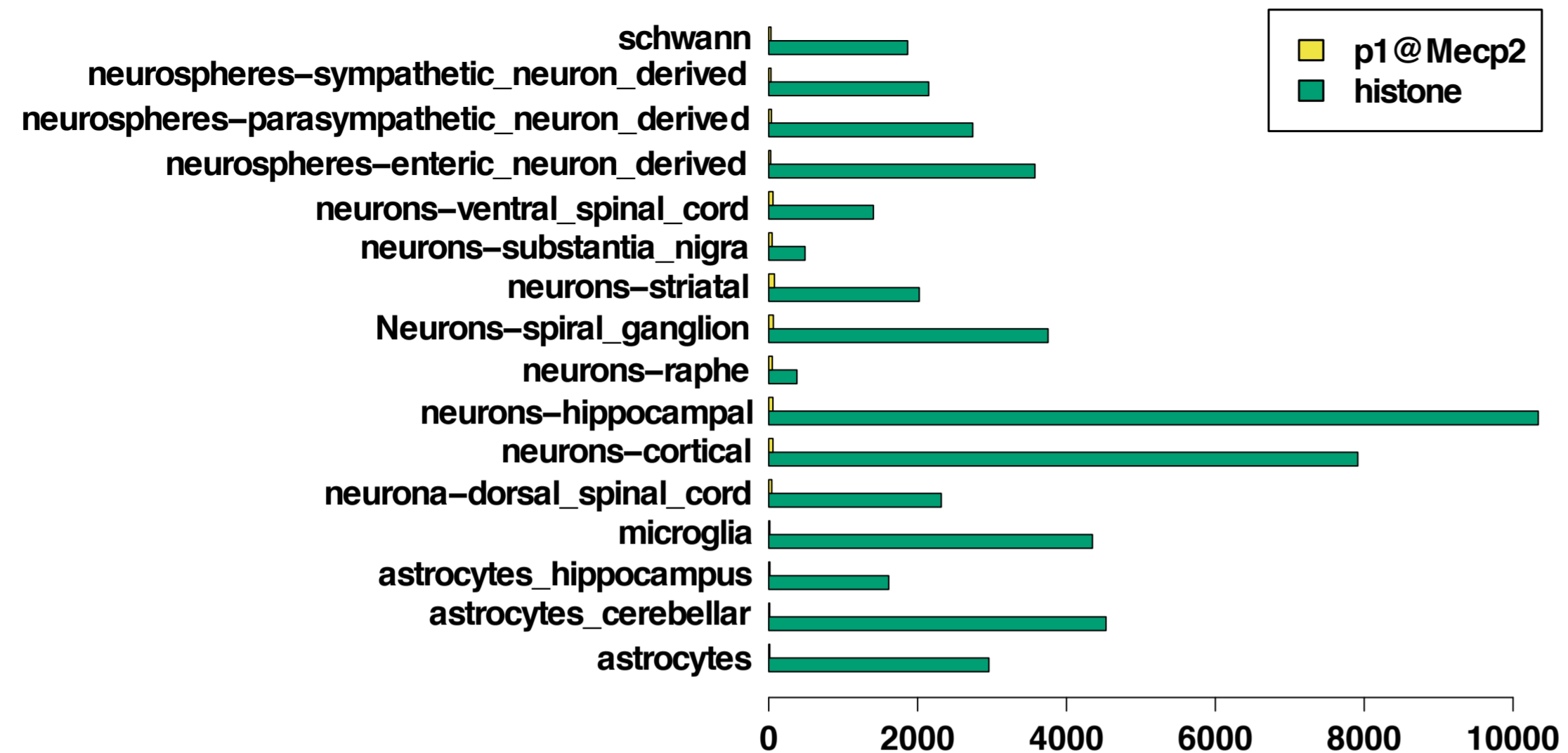

b.

## Mouse tissues

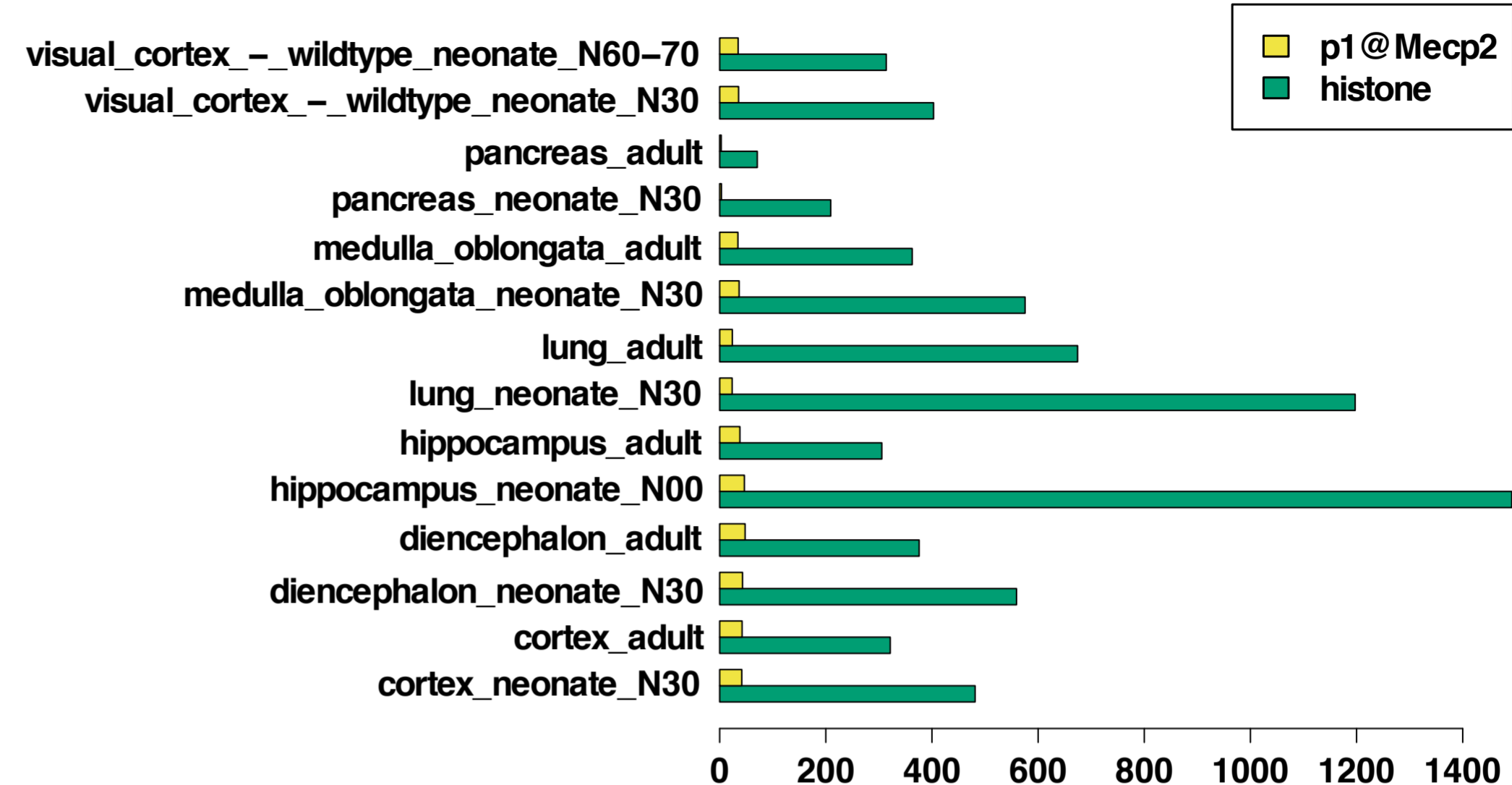

c.

## Human cells

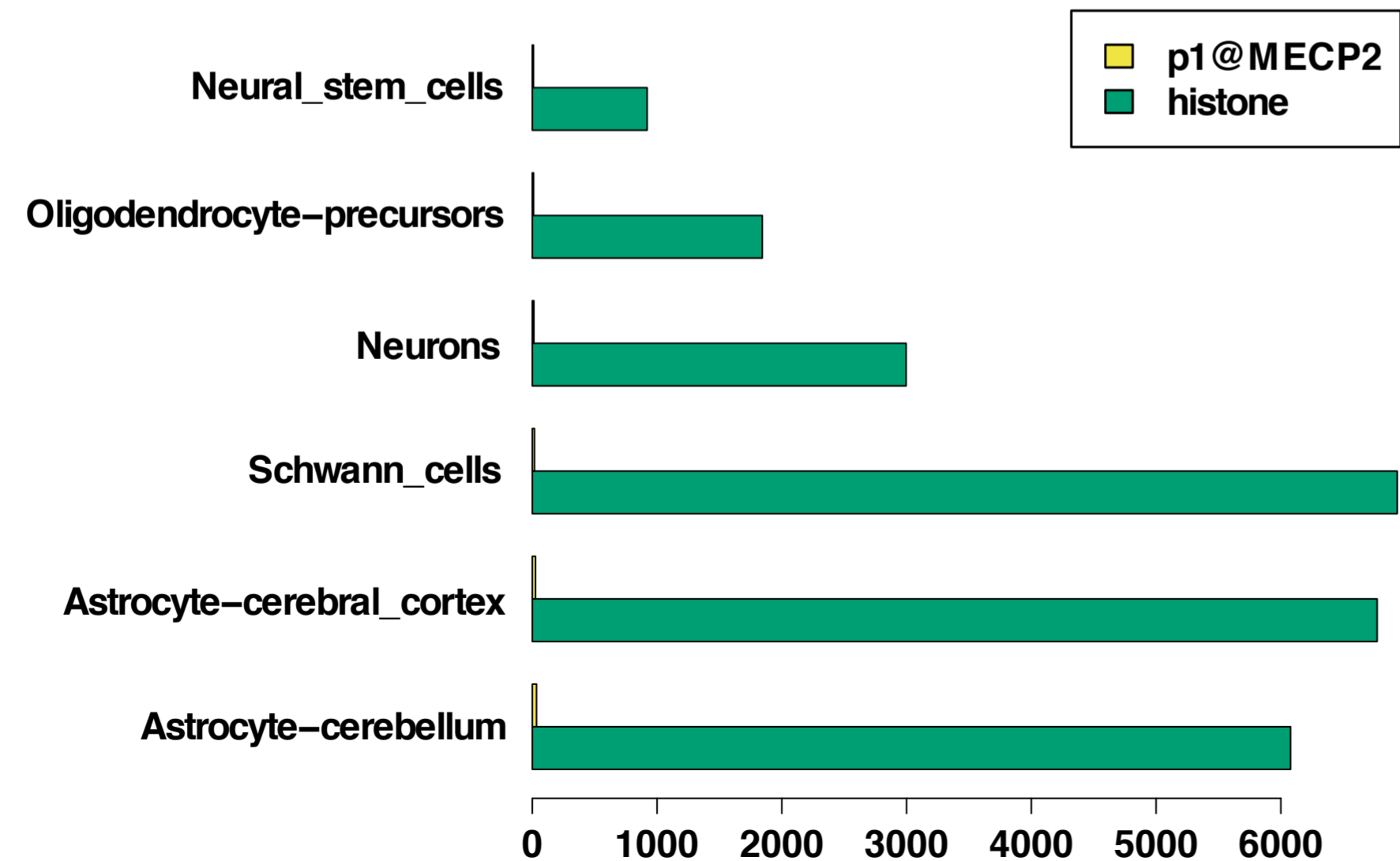

d.

## Human tissues

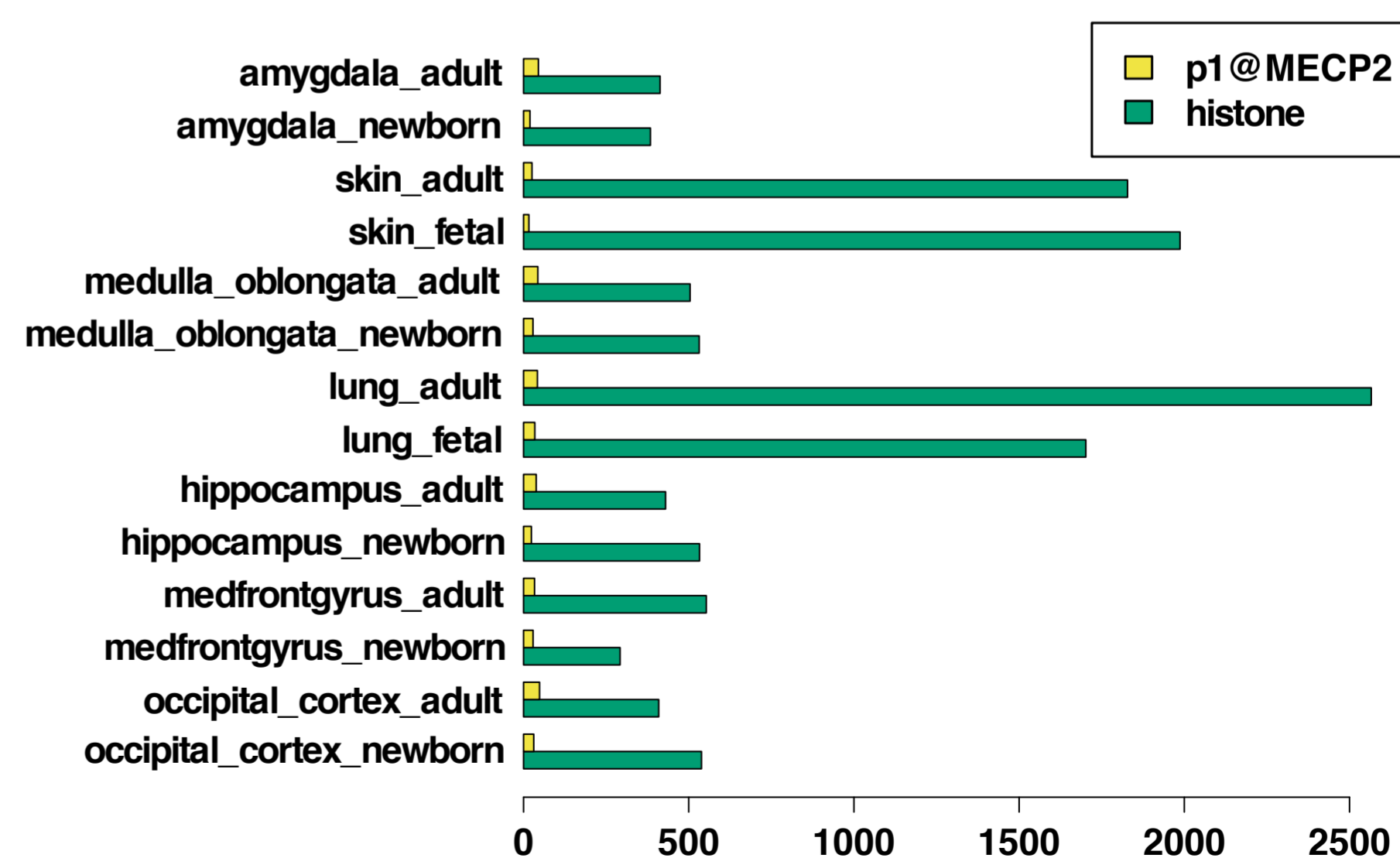

Supplement: Supplementary file 6 — Additional file 6: Figure S5: Developmental profile for the 3 genes in human brain. Human FOXG1 (a), MECP2 (b) and CDKL5 (c) expression in TPM across a set of adult, newborn and fetal brain regions is shown as labeled. FOXG1 shows the highest overall expression as well as having higher expression in fetal than in adult samples as opposed to the expression of MECP2 and CDKL5 in the same samples. (PDF 39 KB) [file 12864_2013_7082_MOESM6_ESM.pdf]

a.

## Mouse cerebellum

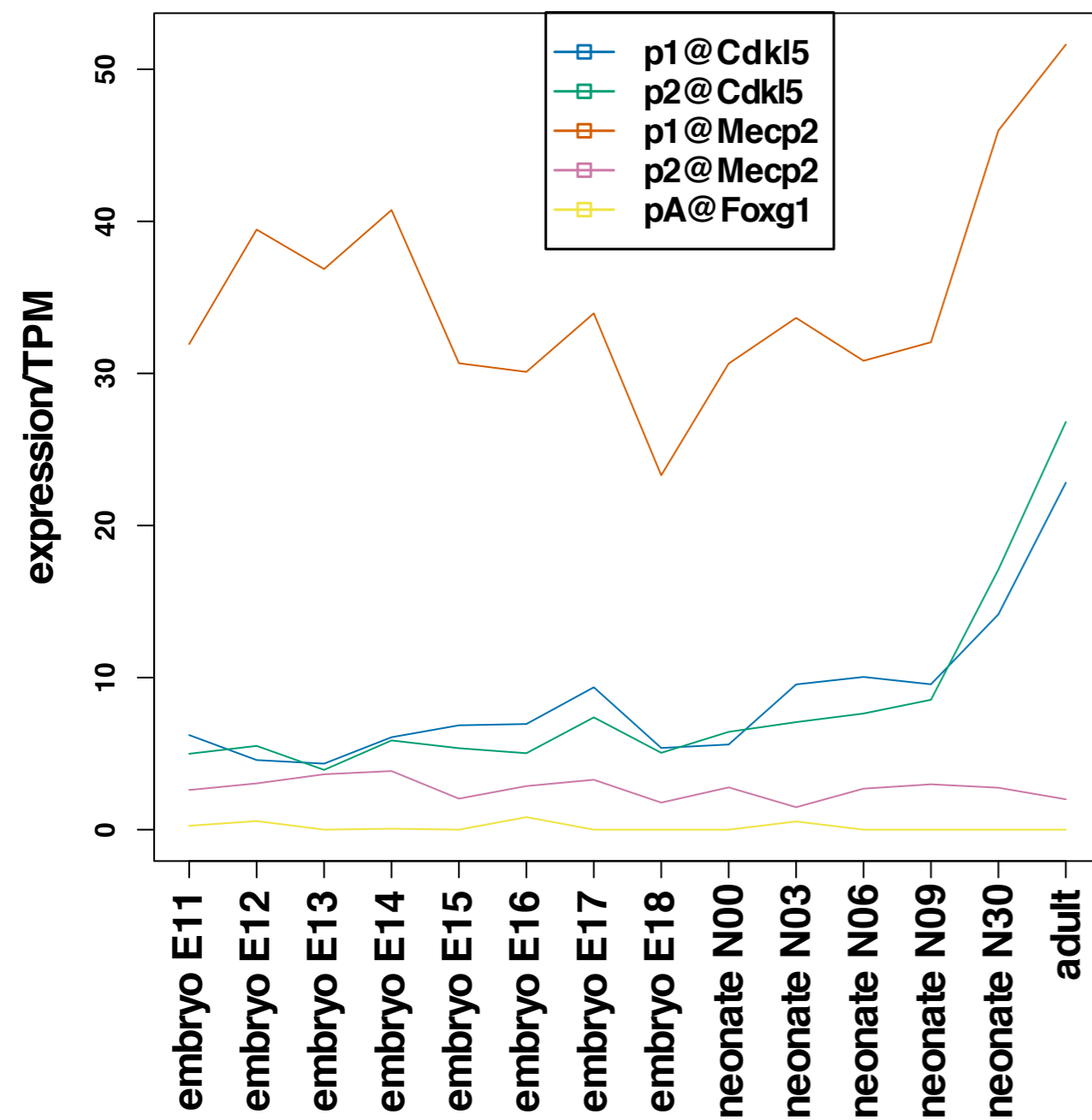

b.

## Mouse visual cortex

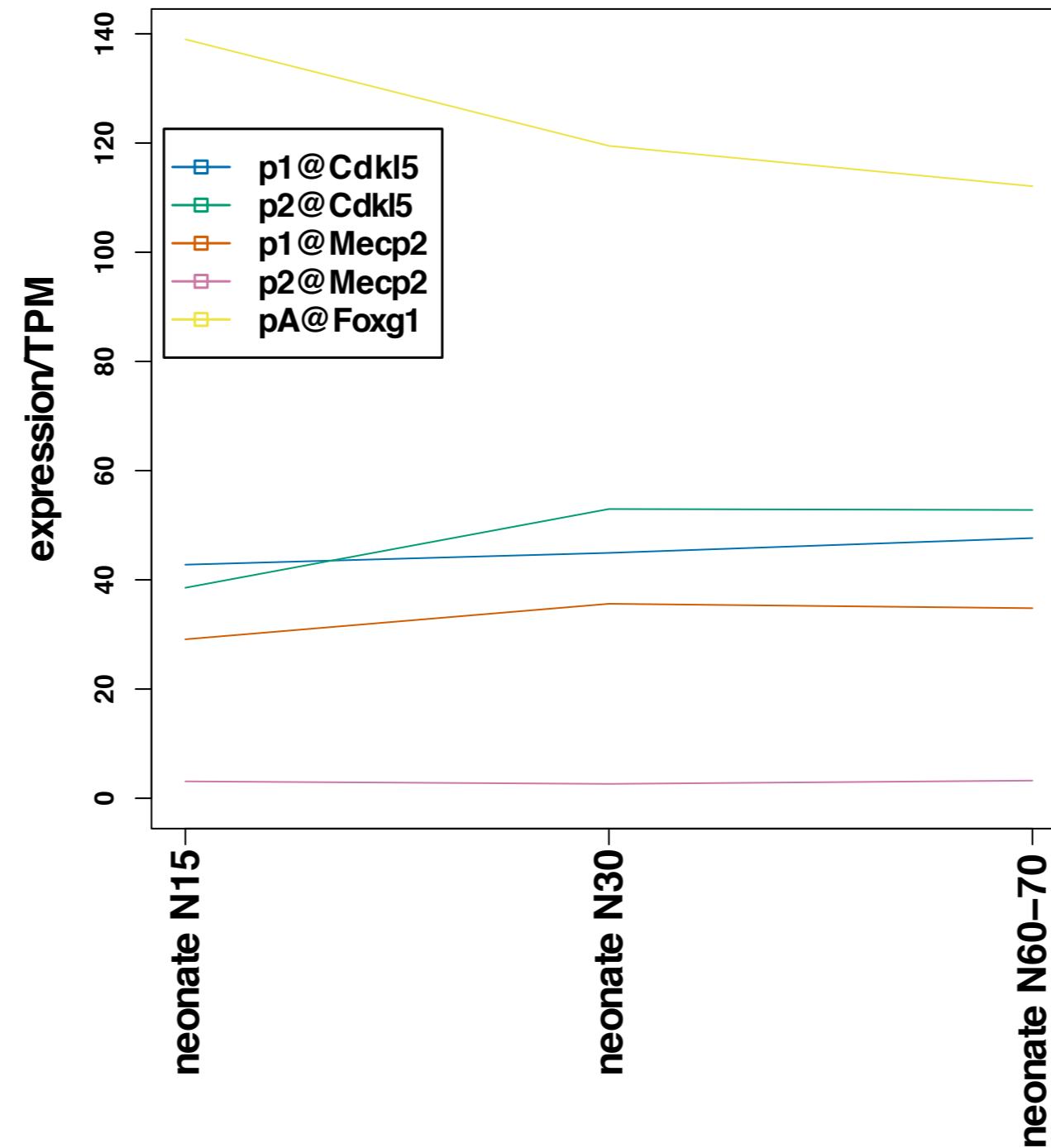

c.

## Mouse pituitary gland

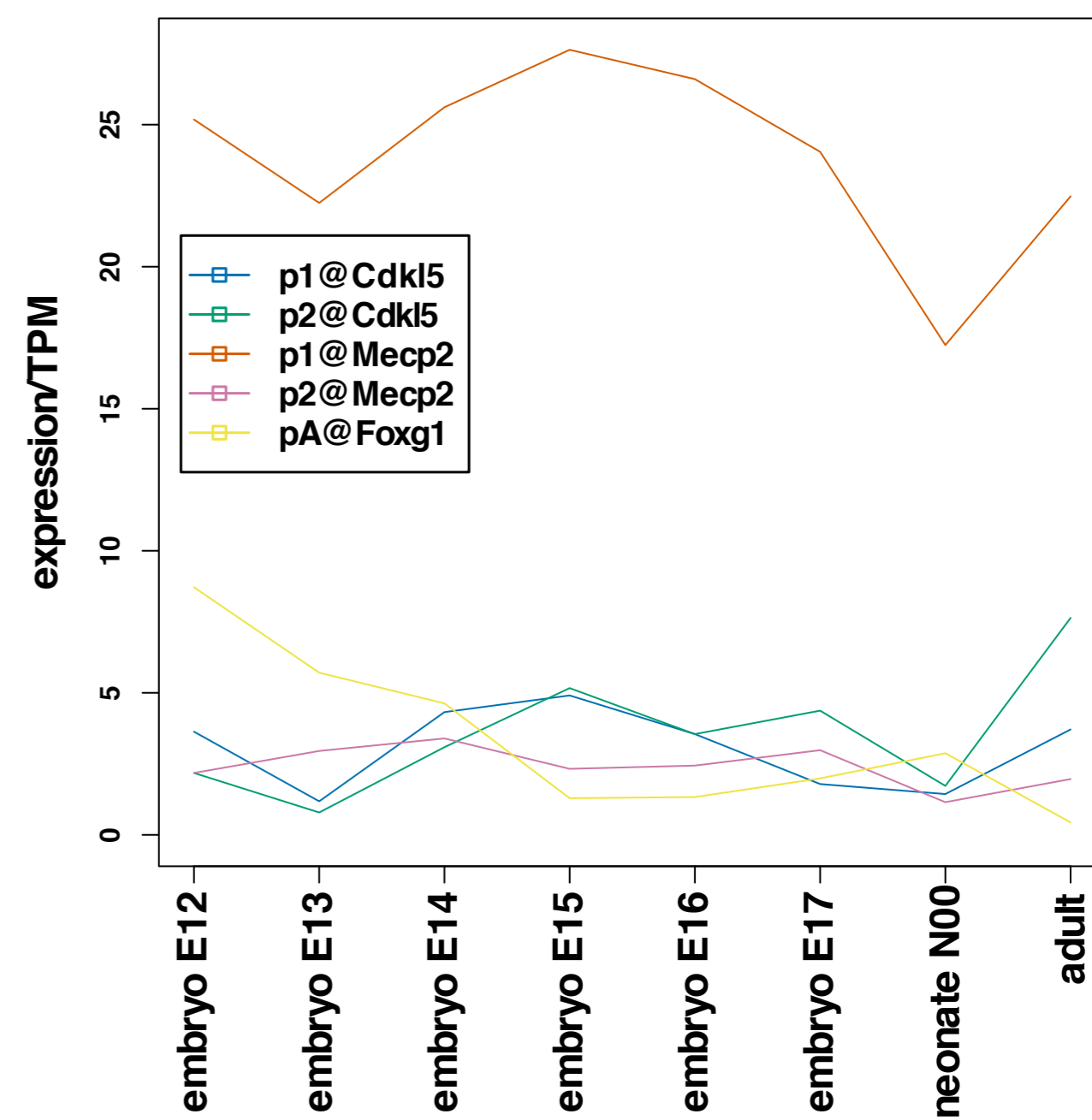

Supplement: Supplementary file 7 — Additional file 7: Figure S4: Expression profile of the three genes in mouse in developing brain tissues. Line plots showing expression of selected promoters of Foxg1, Mecp2 and Cdkl5 during development in mouse cerebellum (panel a), mouse visual cortex (panel b) and mouse pituitary gland (panel c). Refer main text for details. (PDF 41 KB) [file 12864_2013_7082_MOESM7_ESM.pdf]

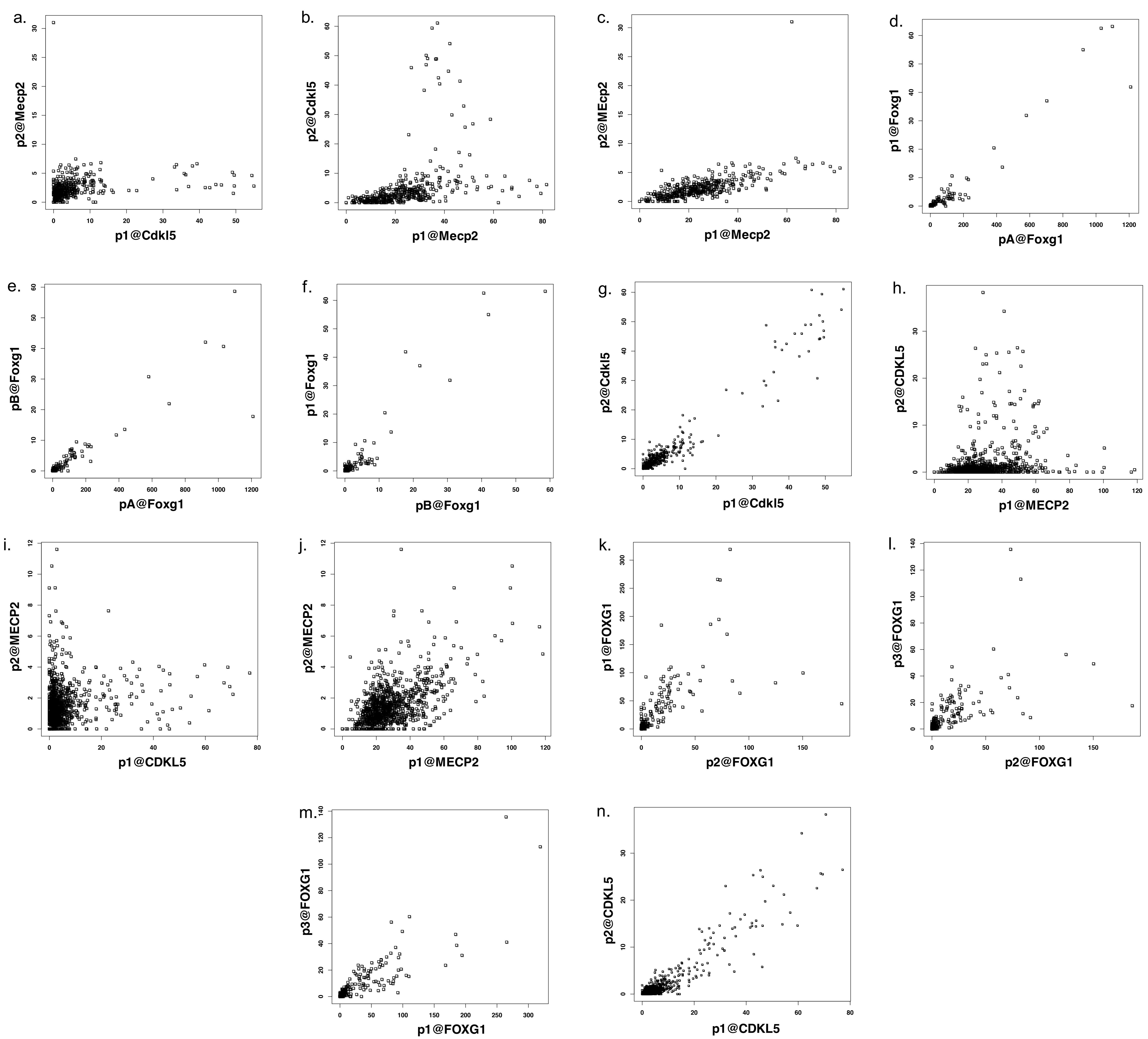

Supplement: Supplementary file 11 — Additional file 11: Figure S7: Intra and inter gene expression correlations between the three genes. Expression correlation plots for all other promoter combinations not present in Figure 3. Plots a-g are mouse promoters, while plots h-n are human promoters as labeled. (PDF 495 KB) [file 12864_2013_7082_MOESM11_ESM.pdf]
