# Supplementary material for: CAGE-defined promoter regions of the genes implicated in Rett Syndrome
Source: BMC Genomics. 2014 Dec 24;15(1):1177. doi: 10.1186/1471-2164-15-1177 (PMC4522966; doi:10.1186/1471-2164-15-1177)
Supplement: Supplementary file 15 — Additional file 15: Figure S9: Promoter shapes for all the other promoters. The shapes of all the individual promoters in mouse (a-e) and human (f-m) are shown as labeled. The shapes are drawn from the first nucleotide of the first mapped CAGE tag to the first nucleotide of the last mapped CAGE tag, the y-axis shows the counts in TPM for each position. (PDF 41 KB) [file 12864_2013_7082_MOESM15_ESM.pdf]

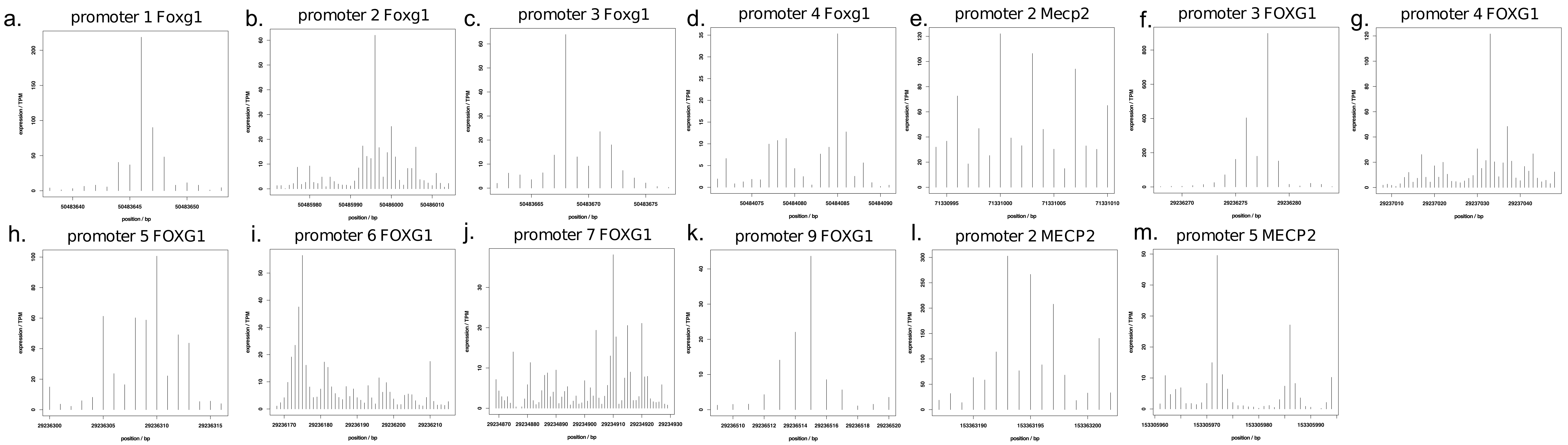
a.
promoter 1 Foxg1
b.
promoter 2 Foxg1
c.
promoter 3 Foxg1
d.
promoter 4 Foxg1
e.
promoter 2 Mecp2
f.
promoter 3 FOXG1
g.
promoter 4 FOXG1
h.
promoter 5 FOXG1
i.
promoter 6 FOXG1
j.
promoter 7 FOXG1
k.
promoter 9 FOXG1
l.
promoter 2 MECP2
m.
promoter 5 MECP2
expression / TPM
position / bp
