# Supplementary material for: CAGE-defined promoter regions of the genes implicated in Rett Syndrome
Source: BMC Genomics. 2014 Dec 24;15(1):1177. doi: 10.1186/1471-2164-15-1177 (PMC4522966; doi:10.1186/1471-2164-15-1177)
Supplement: Supplementary file 16 — Additional file 16: Table S7: List of transcription factors with high binding probability of 0.7 and above to the promoters of the three genes in mouse (A) and human (B) genome. Transcription factors common to the three genes are shown in red. (DOC 48 KB) [file 12864_2013_7082_MOESM16_ESM.doc]

Supplementary Table 7: List of transcription factors with high binding probability of 0.7 and above to the promoters of the three genes in mouse (A) and human (B) genome. Transcription factors common to the three genes are shown in red.

**Supplementary table 7**A

| **FOXG1** | **MECP2** | **CDKL5** |
| --- | --- | --- |
| GTF2I | RREB1 | SP1 |
| MED-1 | YY1 | ZFP161 |
| FOXP1 | DMAP1, NCOR1, NCOR2, SMARC | NFY |
| IRF1,2 | FOXP1 | PAX5 |
| PAX5 | GTF2I | TFAP2A, TFAP2C |
| RREB1 | EGR1, EGR2, EGR3 | FOXP1 |
| ELK1, ELK4, GABPA, GABPB1 | SP1 | RUNX1, RUNX2, RUNX3 |
| NFY | TFAP2B | MED-1 |
| MYF family | NANOG | SOX17 |
| TFAP2B | NFY | CRX |
| NANOG | TOPORS | NFATC1, NFATC2, NFATC.3 |
| TFAP2A, TFAP2C |  | FOXM1 |
| AHR, ARNT, ARNT2 |  | RREB1 |
| REST |  | KLF4 |
| MAFB |  |  |
| MAZ |  |  |
| YY1 |  |  |
| NKX2-2, NKX2-8 |  |  |
| ZBTB6 |  |  |
| TFDP1 |  |  |
| SPI1 |  |  |
| ZNF384 |  |  |
| POU5F1 |  |  |

**Supplementary table 7B**

| **Foxg1** | **Mecp2** | **Cdkl5** |
| --- | --- | --- |
| GTF2I | MAZ | NKX2-2, NKX2-8 |
| IRF1, IRF2 | TFAP2B | SP1 |
| TFDP1 | NANOG | NFY |
| TFAP2B | SP1 |  |
| EGR1, EGR2, EGR3 | NFY |  |
| SP1 |  |  |
| MAZ |  |  |
| NFY |  |  |
| MYF family |  |  |
| REST |  |  |
| NANOG |  |  |
| TFAP2A, TFAP2C |  |  |
| AHR, ARNT, ARNT2 |  |  |
| RREB1 |  |  |
| YY1 |  |  |
| NKX2-2, NKX2-8 |  |  |
| ZBTB6 |  |  |
| SPI1 |  |  |
| ZNF384 |  |  |
| POU5F1 |  |  |
| MAFB |  |  |
