# Supplementary material for: CAGE-defined promoter regions of the genes implicated in Rett Syndrome
Source: BMC Genomics. 2014 Dec 24;15(1):1177. doi: 10.1186/1471-2164-15-1177 (PMC4522966; doi:10.1186/1471-2164-15-1177)
Supplement: Supplementary file 14 — Additional file 14: Figure S8: Locations of active enhancers correlated to the three genes in human samples. UCSC snapshot showing the positions of all eRNA producing human enhancers that are correlated to the expression of the three genes: FOXG1 (a), MECP2 (b) and CDKL5 (c). (PDF 58 KB) [file 12864_2013_7082_MOESM14_ESM.pdf]

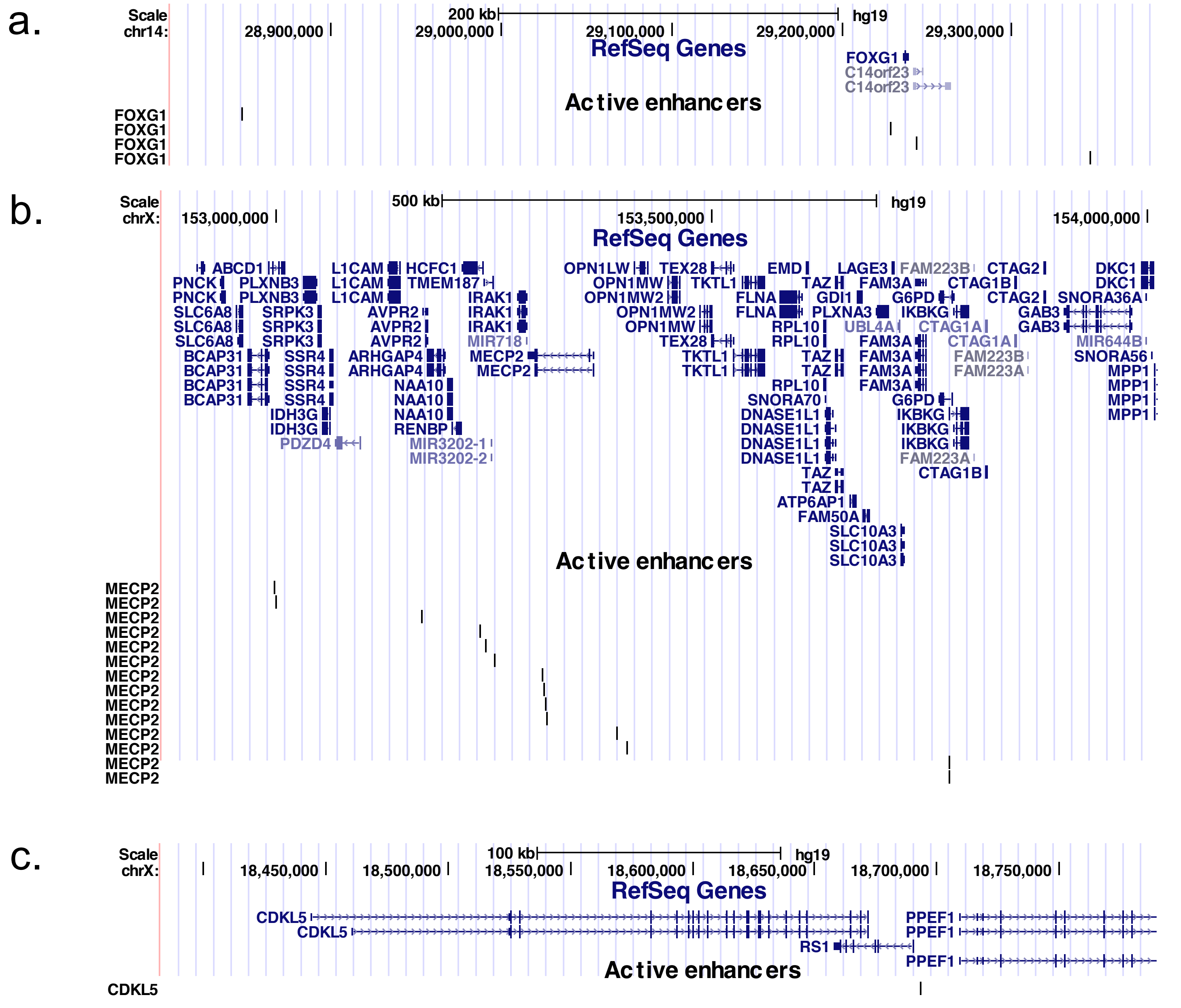
a.
Scale
chr14:
200 kb
hg19
28,900,000
29,000,000
29,100,000
29,200,000
29,300,000
RefSeq Genes
FOXG1
C14orf23
C14orf23
Active enhancers
FOXG1
FOXG1
FOXG1
FOXG1
b.
Scale
chrX:
500 kb
hg19
153,000,000
153,500,000
154,000,000
RefSeq Genes
ABCD1
PNCK
PNCK
SLC6A8
SLC6A8
SLC6A8
BCAP31
BCAP31
BCAP31
BCAP31
PLXNB3
PLXNB3
SRPK3
SRPK3
SRPK3
SSR4
SSR4
SSR4
SSR4
IDH3G
IDH3G
PDZD4
L1CAM
L1CAM
L1CAM
AVPR2
AVPR2
AVPR2
ARHGAP4
ARHGAP4
NAA10
NAA10
NAA10
RENBP
MIR3202-1
MIR3202-2
HCFC1
TMEM187
IRAK1
IRAK1
IRAK1
MIR718
MECP2
MECP2
OPN1LW
OPN1MW
OPN1MW2
OPN1MW2
OPN1MW
TEX28
TEX28
TKTL1
TKTL1
TKTL1
EMD
FLNA
FLNA
RPL10
RPL10
RPL10
SNORA70
DNASE1L1
DNASE1L1
DNASE1L1
DNASE1L1
TAZ
TAZ
TAZ
TAZ
TAZ
ATP6AP1
FAM50A
SLC10A3
SLC10A3
SLC10A3
GDI1
PLXNA3
UBL4A
LAGE3
FAM3A
FAM3A
FAM3A
FAM3A
FAM3A
G6PD
G6PD
IKBKG
IKBKG
IKBKG
IKBKG
FAM223B
FAM223A
CTAG1A
CTAG1A
FAM223B
FAM223A
CTAG1B
CTAG2
CTAG1B
CTAG2
GAB3
GAB3
MIR644B
DKC1
DKC1
SNORA36A
SNORA56
MPP1
MPP1
MPP1
MPP1
Active enhancers
MECP2
MECP2
MECP2
MECP2
MECP2
MECP2
MECP2
MECP2
MECP2
MECP2
MECP2
MECP2
MECP2
MECP2
c.
Scale
chrX:
100 kb
hg19
18,450,000
18,500,000
18,550,000
18,600,000
18,650,000
18,700,000
18,750,000
RefSeq Genes
CDKL5
CDKL5
RS1
PPEF1
PPEF1
PPEF1
Active enhancers
CDKL5
